# Artificial intelligence-assisted double reading of chest radiographs to detect clinically relevant missed findings: a two-centre evaluation

**DOI:** 10.1007/s00330-024-10676-w

**Published:** 2024-03-11

**Authors:** Laurens Topff, Sanne Steltenpool, Erik R. Ranschaert, Naglis Ramanauskas, Renee Menezes, Jacob J. Visser, Regina G. H. Beets-Tan, Nolan S. Hartkamp

**Affiliations:** 1 Department of Radiology, Netherlands Cancer Institute, Amsterdam, The Netherlands; 2 GROW School for Oncology and Reproduction, Maastricht University, Maastricht, The Netherlands; 3 Department of Radiology and Nuclear Medicine, Erasmus MC, University Medical Center Rotterdam, Rotterdam, The Netherlands; 4 Department of Radiology, Elisabeth-TweeSteden Hospital, Tilburg, The Netherlands; 5 Department of Radiology, St. Nikolaus Hospital, Eupen, Belgium; 6 Ghent University, Ghent, Belgium; 7 Oxipit UAB, Vilnius, Lithuania; 8 Department of Radiology, Nuclear Medicine and Medical Physics, Institute of Biomedical Sciences, Faculty of Medicine, Vilnius University, Vilnius, Lithuania; 9 Biostatistics Centre, Department of Psychosocial Research and Epidemiology, Netherlands Cancer Institute, Amsterdam, The Netherlands

**Keywords:** Thoracic radiography, Diagnostic errors, Artificial intelligence, Natural language processing, Healthcare quality assurance

## Abstract

**Objectives:**

To evaluate an artificial intelligence (AI)–assisted double reading system for detecting clinically relevant missed findings on routinely reported chest radiographs.

**Methods:**

A retrospective study was performed in two institutions, a secondary care hospital and tertiary referral oncology centre. Commercially available AI software performed a comparative analysis of chest radiographs and radiologists’ authorised reports using a deep learning and natural language processing algorithm, respectively. The AI-detected discrepant findings between images and reports were assessed for clinical relevance by an external radiologist, as part of the commercial service provided by the AI vendor. The selected missed findings were subsequently returned to the institution’s radiologist for final review.

**Results:**

In total, 25,104 chest radiographs of 21,039 patients (mean age 61.1 years ± 16.2 [SD]; 10,436 men) were included. The AI software detected discrepancies between imaging and reports in 21.1% (5289 of 25,104). After review by the external radiologist, 0.9% (47 of 5289) of cases were deemed to contain clinically relevant missed findings. The institution’s radiologists confirmed 35 of 47 missed findings (74.5%) as clinically relevant (0.1% of all cases). Missed findings consisted of lung nodules (71.4%, 25 of 35), pneumothoraces (17.1%, 6 of 35) and consolidations (11.4%, 4 of 35).

**Conclusion:**

The AI-assisted double reading system was able to identify missed findings on chest radiographs after report authorisation. The approach required an external radiologist to review the AI-detected discrepancies. The number of clinically relevant missed findings by radiologists was very low.

**Clinical relevance statement:**

The AI-assisted double reader workflow was shown to detect diagnostic errors and could be applied as a quality assurance tool. Although clinically relevant missed findings were rare, there is potential impact given the common use of chest radiography.

**Key Points:**

*• A commercially available double reading system supported by artificial intelligence was evaluated to detect reporting errors in chest radiographs (n=25,104) from two institutions.*

*• Clinically relevant missed findings were found in 0.1% of chest radiographs and consisted of unreported lung nodules, pneumothoraces and consolidations.*

*• Applying AI software as a secondary reader after report authorisation can assist in reducing diagnostic errors without interrupting the radiologist’s reading workflow. However, the number of AI-detected discrepancies was considerable and required review by a radiologist to assess their relevance.*

**Graphical Abstract:**

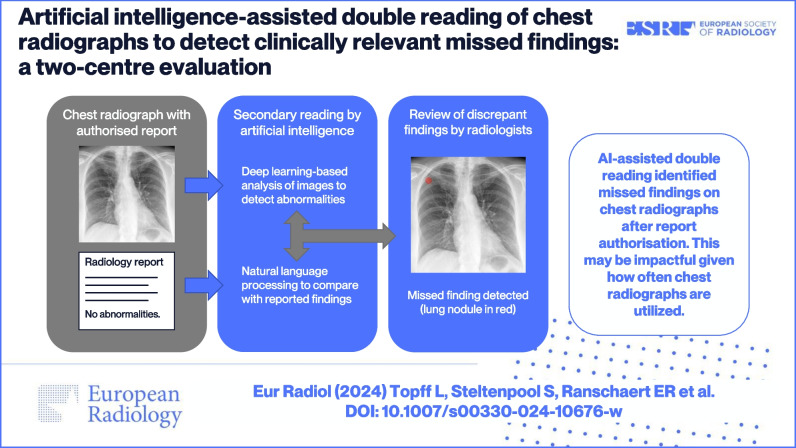

**Supplementary Information:**

The online version contains supplementary material available at 10.1007/s00330-024-10676-w.

## Introduction

Chest radiography continues to be one of the most frequently performed radiological examinations worldwide [1]. It is the first-line imaging modality for the diagnosis and follow-up of many cardiothoracic diseases due to its wide availability, low cost and low radiation exposure. Although the reporting of chest radiographs is part of the daily work for many radiologists, its interpretation can remain challenging even for experienced readers. Chest radiographs are known to have blind spots as a consequence of being two-dimensional projections of three-dimensional structures. The interpretation is prone to frequent errors due to missed findings such as lung nodules, pneumonia and pneumothorax [2].

The miss rate in conventional radiography was previously investigated and is highly dependent on study design and setting [3]. In studies with a mixture of normal and abnormal cases similar to daily clinical practice, disagreement rates of 1–3% were found amongst radiologists [4, 5]. An unreported finding on chest radiography can result in a missed or delayed diagnosis. In addition, diagnostic errors may lead to medicolegal issues. Missed lung cancer is one of the most frequent reasons for malpractice actions against radiologists [6, 7]. Early detection of lung cancer can significantly affect the treatment options and prognosis of patients [8, 9]. The clinical relevance of other missed radiological findings is less studied; however, it is clear that certain undetected findings, such as device malposition and pneumothorax, can negatively impact patient outcomes as well.

Double reading of imaging examinations by peers is a common practice in several countries to reduce diagnostic errors and improve the quality of reports [10, 11]. However, peer review is a time-consuming task and previously reported discrepancy rates between readers were rather low [12]. The cost-effectiveness of double reading systems should therefore be considered, especially for high-volume, low-complexity examinations such as chest radiography.

The use of artificial intelligence (AI) solutions can be another method to mitigate the risk of diagnostic errors. Multiple studies have demonstrated the ability of deep learning models to classify findings on chest radiographs, and several commercial AI applications have been developed for this purpose [13, 14]. Radiologists assisted by these tools have shown improved detection performances for a variety of findings, such as lung nodules, pneumonia and pneumothorax [15–21]. Although these studies demonstrate beneficial effects on sensitivity, implementing AI software as a concurrent reader in clinical practice may lead to an increase in the radiologist’s workload [22]. AI results that are not seamlessly integrated into the workflow or contain false-positive findings can disrupt the radiologist’s reading. Recent validation studies on commercial AI applications for chest radiography reported an increase in interpretation times [23–25], or showed only limited reading time savings of seconds [19, 20, 26, 27]. A clear lack of efficiency gains will likely hinder the widespread adoption of these tools.

However, there is potential for current AI applications to support the double reading of imaging examinations. In this scenario, AI software is applied as a secondary reader to identify errors in the radiologist’s interpretation after the report has been signed off. This approach uses a combination of AI-based image analysis and natural language processing (NLP) of reports to detect discrepancies. The ability of NLP models to accurately classify findings in reports of chest radiographs has previously been demonstrated [28]. The double reader approach has the benefit of not interrupting the radiologist’s primary reading workflow. Only a fraction of examinations with discrepant findings needs to be reviewed.

In this study, we aim to evaluate an AI-assisted double reading system to identify clinically relevant missed findings on routinely reported chest radiographs. We investigate the applicability in two distinct settings, a general hospital and a tertiary oncology centre.

## Materials and methods

### Study design

A retrospective observational study was performed in two institutions: a secondary care general hospital, Elisabeth-TweeSteden Hospital, Tilburg, Netherlands (institution 1), and a tertiary referral centre, the Netherlands Cancer Institute, Amsterdam, Netherlands (institution 2). The study was approved by the ethics committee of Institution 1 (NW2021-57) and the Institutional Review Board of Institution 2 (IRBd19163). The requirement for written informed consent was waived due to the retrospective nature of the study.

**Fig. 1:**
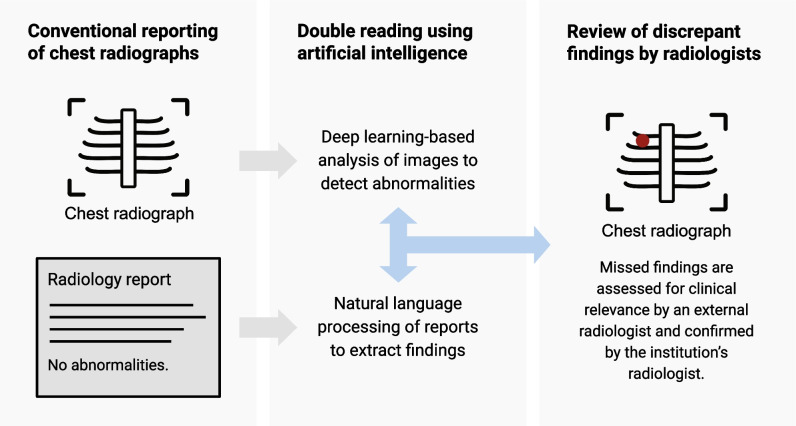
Artificial intelligence assisted double reading workflow of chest radiographs

The double reading system in this validation study consisted of an AI-based software application that performed a comparative analysis of chest radiographs and corresponding radiology reports, and subsequently, a review by radiologists (Fig. 1).

### Patient and imaging selection

Adult patients (≥ 18 years) who underwent chest radiography were consecutively selected from April 2021 to February 2022 in institution 1, and from January to December 2018 in institution 2. The AI software analysis was limited to chest radiographs with posteroanterior (PA) projection; therefore, bed-side chest radiographs were not included. Images and corresponding radiology reports were transferred from the picture archiving and communication system (PACS) to the AI pipeline for analysis, via a local implementation in institution 1 and remotely for institution 2. The data were analysed using available metadata regarding projection and patient age. Chest radiographs acquired with anteroposterior (AP) projection were excluded.

### Artificial intelligence analysis

The investigated AI software (ChestEye Quality, Oxipit) was CE (Conformité Européenne) marked as a class IIb medical device and commercially available. The software processed both chest radiographs and corresponding reports to identify missed findings. Chest radiographs were analysed by a deep learning algorithm that was initially trained to detect multiple radiological findings. The algorithm was repurposed for use in the double reading system, thereby focusing only on findings that were considered potentially clinically actionable when missed. The key pathologies that could trigger further review were as follows: nodule/mass, consolidation, pneumothorax, pneumomediastinum, pneumoperitoneum, rib fractures and device malposition. Other findings (e.g., aortic sclerosis, azygos lobe, spinal degenerative changes) could not prompt further review. The software calculated the probability of each finding. In addition, a heatmap was generated to highlight the detected abnormalities as an overlay on the original image. This was done for explainability purposes and facilitated the reader to verify the AI result. Secondly, the software analysed the corresponding text reports of radiologists using a rule-based NLP algorithm (ChestEye Quality, Oxipit), thereby extracting reported findings in a structured manner. Using the obtained information, the software automatically compared imaging and reported findings to assess discrepancies that could represent missed findings.

### Review

The AI-detected discrepancies were listed in a web-based analytics platform for further review (Supplementary Figure S1). The first review was part of the commercially available service provided by the AI vendor (Oxipit) and was performed by an external radiologist (5 years of experience) who was employed by the vendor. The dedicated platform enabled the external radiologist to review cases in batches. Using imaging, radiology reports, AI findings and generated heatmaps, the reviewer categorised each examination in a binary manner: (1) no missed findings in the radiology report or missed findings that are not clinically relevant, (2) missed findings that are clinically relevant. Clinically relevant missed findings were defined as findings that have the potential to change the diagnosis and patient management, and adversely affect the patient’s outcome when unreported. The external radiologist did not have access to the patient’s prior imaging examinations. In order to facilitate an efficient review, the external radiologist provided a brief assessment only for cases with clinically relevant missed findings and not for rejected cases. Subsequently, the selection of clinically relevant missed findings was returned to the institutions for internal review. The radiologist of each institution (respectively N.H. with 5 years of experience for institution 1, and L.T. with 5 years of experience for institution 2) reviewed the cases for clinical relevance using the same analytics platform, thereby confirming or rejecting the AI-assisted secondary reading result.

### Statistical analysis

The data of both institutions were compared using a chi-square test or Fisher’s exact test, as appropriate. The null hypothesis was rejected when *p* < 0.05. Statistical analyses were performed by a statistician (R.M.) using the R environment for Statistical Computing [29].

## Results

**Fig. 2:**
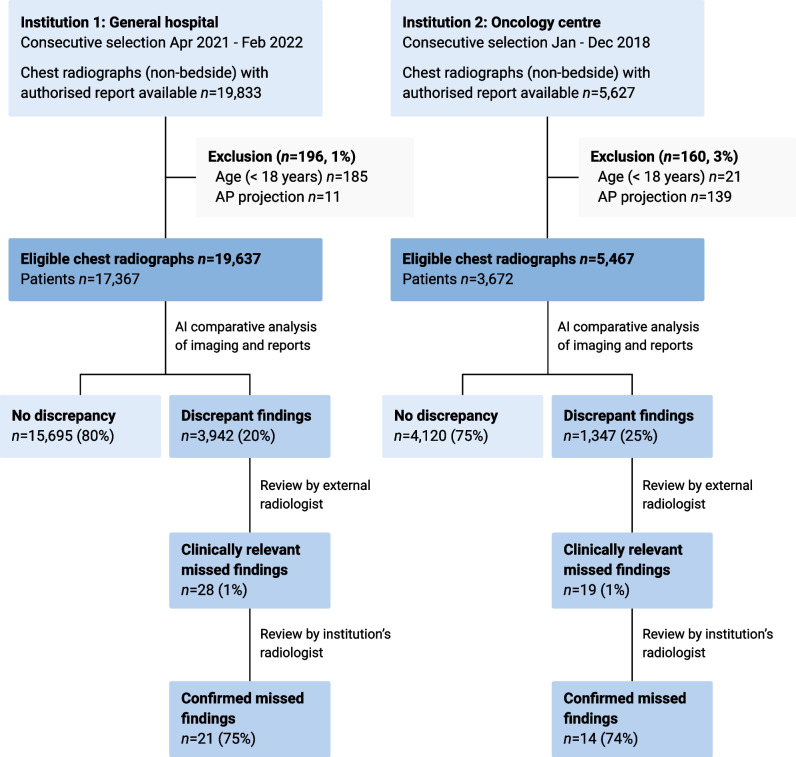
Data flowchart per institution. AP, anteroposterior

A total sample of 25,104 chest radiographs was included from 21,039 unique patients (mean age 61.1 years ± 16.2 [SD]; 10,436 men). The distribution of chest radiographs was 19,637 (78.2%) and 5467 (21.8%) for institutions 1 and 2, respectively. Patient demographics per institution are available in Table 1. The AI software excluded 1.4% (356/25,460) radiographs due to age restriction or AP projection. All remaining cases were successfully analysed by the software. A detailed data flowchart per institution is shown in Fig. 2.

**Table 1:** Patient demographics

	Institution 1: general hospital	Institution 2: oncology centre
Chest radiographs (*n*)	19,637	5467
Patients (*n*)	17,367	3672
Age ± SD (year)	61.1 ± 16.6	61.0 ± 14.0
Male	8744 (50.3%)	1692 (46.1%)
Female	8623 (49.7%)	1980 (53.9%)

*SD* standard deviation

### Natural language processing

The unstructured text reports of chest radiographs were analysed using an NLP algorithm to extract the reported findings as standardised labels. The proportion of cases reported as normal vs. abnormal was different between centres (chi-square test, *p* < 0.001). Reports were classified as normal in 69.5% (13,646/19,637) for institution 1, and in 50.6% (2768/5467) for institution 2. The reports with abnormal findings from institution 1 (30.5%, 5991/19,637) were labelled with consolidation in 24.4% (*n*=1463), nodule/mass in 9.2% (*n*=552) and pneumothorax in 1.6% (*n*=93). In contrast, the reports with abnormal findings from institution 2 (49.4%, 2699/5467) were labelled with consolidation in 29.9% (*n*=806), and showed a higher number of nodule/mass in 24.9% (*n*=672), and pneumothorax in 11.0% (*n*=297).

### Deep learning–based image processing

The chest radiographs were analysed by the AI software to detect and localise abnormalities. The image analysis results were then automatically compared with the NLP-derived labels. In the majority of cases, 79.9% and 75.4% for institutions 1 and 2, respectively, no missed findings were identified in the radiology reports. In the remaining cases, there was a discordance between the AI imaging analysis and the report analysis (20.1%, 3942/19,637 and 24.6%, 1347/5467 for the respective institutions). The proportion of discrepancies was statistically different between institutions (Pearson’s chi-square test, *p* < 0.001). Table 2 shows the type of discrepant findings, with nodular opacities marked most frequently as a potentially missed finding. Consequently, examinations with discrepant findings were flagged for manual review.

**Table 2:** Discrepant findings after artificial intelligence analysis of chest radiographs and corresponding reports

	Institution 1: general hospital	Institution 2: oncology centre
Nodule/mass	2822 (65.9%)	1047 (68.2%)
Consolidation	1333 (31.1%)	424 (27.6%)
Pneumothorax	108 (2.5%)	61 (4.0%)
Device malposition	21 (0.5%)	4 (0.3%)

More than one finding per examination can be present

### Review

The AI software found discrepancies in 5289 of 25,104 cases (21.1%). All discordant cases were reviewed by the external radiologist, who identified a total of 47 examinations with clinically relevant unreported findings (Table 3). The selected cases consisted of 0.9% (47/5289) of all discordant cases, corresponding to 0.2% of all chest radiographs (47/25,104). The majority of AI-detected discrepancies (99.1%, 5242/5289) were deemed not clinically relevant or were attributed to incorrectly identified findings made by the AI software. For example, radiology reports with less descriptive language were flagged by the AI software because not all imaging findings were explicitly documented in the report. Upon analysing a random sample of 10% of reports (529/5289), we found that 17.8% (94/529) contained less specific terminology such as ‘known extensive disease’, ‘post-radiation effects’ or ‘congestion’. None of the reports were entirely without descriptive details. The review by the external radiologist was performed in less than 5 h using the custom analytics platform, with a mean review time of approximately 3 s per case. The mean review time was low since most discrepancies were instantly identified as false and dismissed in the platform. Only a minority of cases required a longer review time.

**Table 3:** Clinically relevant missed findings on chest radiographs after review by the external radiologist

	Institution 1: general hospital	Institution 2: oncology centre
Nodule/mass	18 (64.3%)	10 (52.6%)
Consolidation	6 (21.4%)	3 (15.8%)
Pneumothorax	4 (14.3%)	6 (31.6%)
Device malposition	0 (0%)	0 (0%)
Total	28	19

Finally, the results were returned to the respective institutions for review by an internal radiologist. The institution’s radiologists confirmed clinically relevant missed findings in 21 out of 28 cases (75.0%) for institution 1, and 14 out of 19 cases (73.7%) for institution 2. The remainder of results (12/47, 25.5%) were rejected by the institution’s radiologists because the finding was not unequivocally present (*n*=7), was known and unchanged in comparison to prior imaging (*n*=3) or was deemed not clinically relevant (*n*=2). Overall, relevant missed findings were found in 0.1% (21/19,637) and 0.3% (14/5467) of all included chest radiographs for the respective institutions. The difference was statistically significant (Pearson’s chi-square test, *p* < 0.05).

**Fig. 3:**
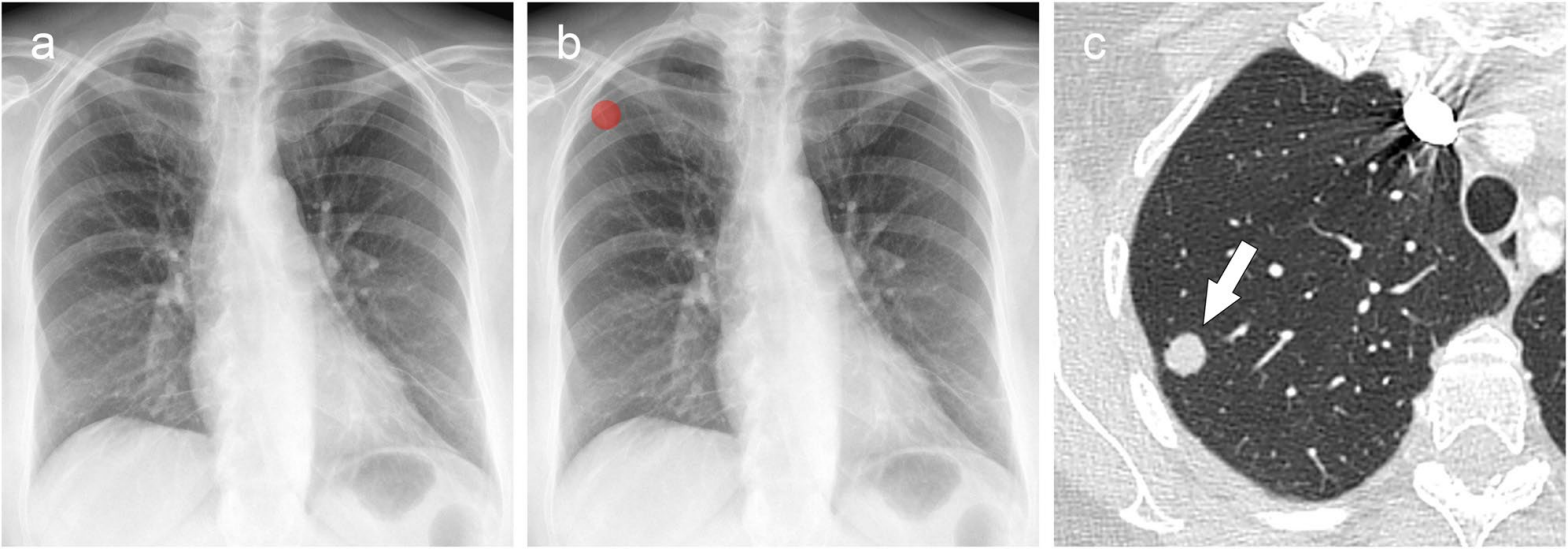
Missed lung nodule on chest radiograph was detected by AI-assisted double reading. **a** Frontal chest radiograph of a 63-year-old patient with ovarian cancer was performed for staging and reported as normal. **b** The AI software detected a nodular opacity in the right upper lung lobe, marked with a heatmap (red). The AI-detected finding was discordant with the radiologist’s report, resulting in the case to be flagged for review. **c** Axial CT image confirmed the presence of a solitary lung lesion (arrow), which was diagnosed as a metastasis of ovarian cancer. AI, artificial intelligence

**Fig. 4:**
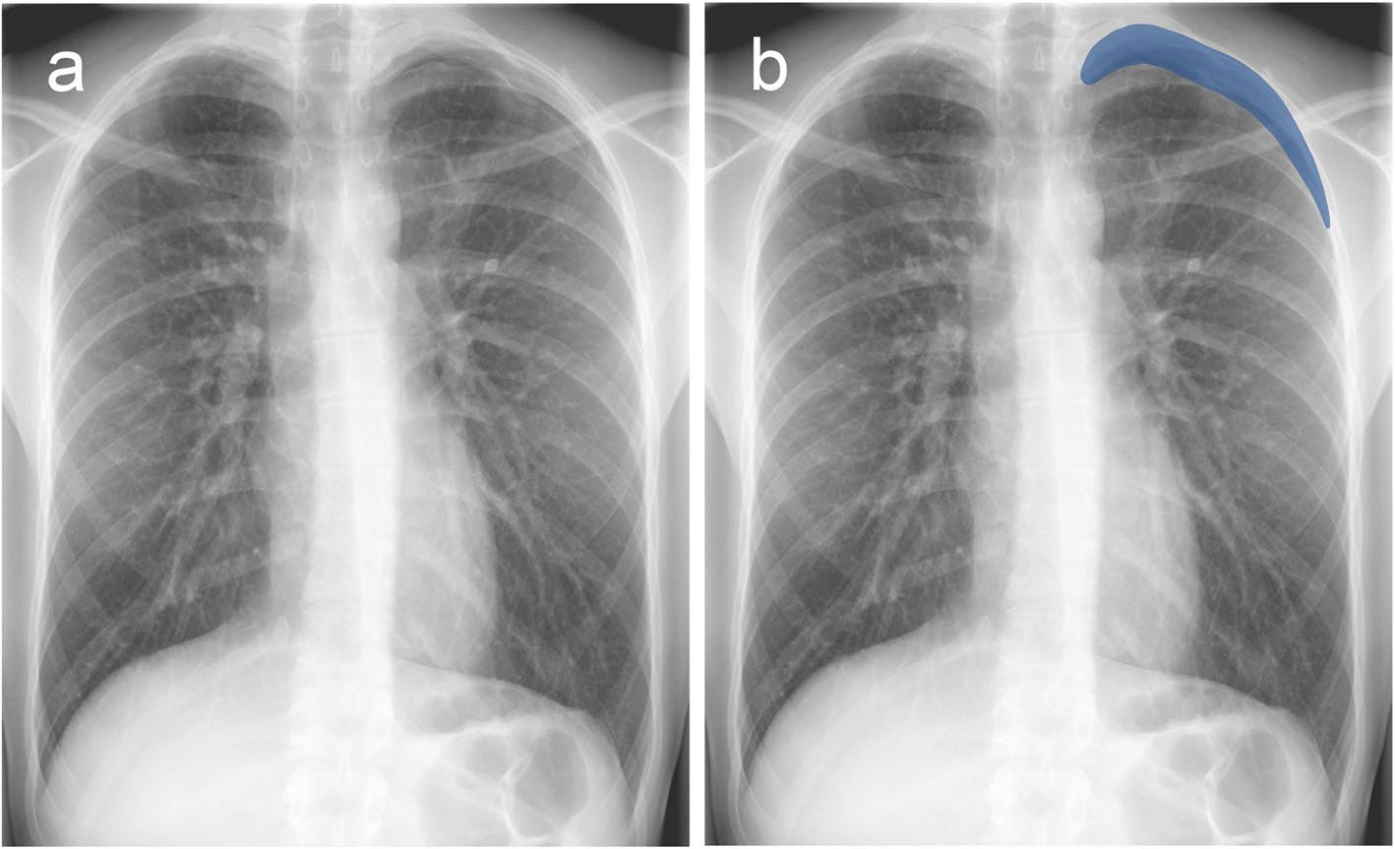
Missed pneumothorax on chest radiograph was detected by AI-assisted double reading. **a** Frontal chest radiograph of a 20-year-old male patient with chest pain after a previous COVID-19 infection. The radiograph was reported as normal. **b** The AI software detected a left apical lucency (blue) consistent with a small apical pneumothorax, which was initially missed by the radiologist. AI, artificial intelligence; COVID-19, coronavirus disease 2019

The list of confirmed unreported findings with clinical significance is shown in Table 4. There was no evidence of difference in the proportion of these findings between institutions (Fisher’s exact test, *p* = 0.6–1). The majority were missed nodular opacities in both institutions, with an example shown in Fig. 3. This resulted in an extra 2.6% (15/567) and 1.5% (10/682) of nodules found for the respective institutions, based on the report analysis. The size of missed nodular opacities was <1 cm in 12 cases and ≥1 cm in 13 cases. The second most frequently unreported finding with clinical significance was pneumothorax. The six missed pneumothoraces were limited to apical lucencies (<3 cm). An example of a missed pneumothorax is shown in Fig. 4. A detailed list of missed findings with chest radiographs, reports, AI-generated results and radiologist’s assessments is available in the Supplementary Material (Appendix A, electronic supplemental material).

**Table 4:** Confirmed clinically relevant findings on chest radiographs after review by the institution’s radiologists

	Institution 1: general hospital	Institution 2: oncology centre
Nodule/mass	15 (71.4%)	10 (71.4%)
Consolidation	3 (14.3%)	1 (7.1%)
Pneumothorax	3 (14.3%)	3 (21.4%)
Device malposition	0 (0%)	0 (0%)
Total	21	14

## Discussion

To our knowledge, this is the first study to investigate an AI-assisted double reading workflow for chest radiography. The study was performed retrospectively on a large cohort (*n* = 25,104) from two institutions, a general hospital and a tertiary oncology centre. A clinically certified AI tool was repurposed as a second reader to identify missed findings on routinely reported chest radiographs. The AI software detected discrepant findings in 21.1% of cases (5289 of 25,104). Therefore, an external radiologist, provided by the AI vendor as part of the commercially available service, reviewed the discrepancies and found clinically relevant missed findings in 0.9% (47 of 5289). The institutions’ radiologists confirmed 35 of 47 missed findings (74.5%) as clinically relevant (0.1% of all cases). The missed findings consisted predominantly of undetected lung nodules, but also included pneumothoraces and consolidations. Although there was a higher percentage of abnormal chest radiographs in the cohort of the oncology centre versus the general hospital, the type of missed findings was similar between both institutions.

The AI-assisted double reading system could be used as a quality assurance tool for the reporting of chest radiographs. While the percentage of clinically relevant missed findings was very low, the potential clinical impact of reducing reporting errors can be considerable in institutions with a high volume of chest radiography. Identifying chest radiographs with missed lung nodules can prevent delayed diagnosis of primary lung cancers or inaccurate staging of cancer patients. Similarly, avoiding a missed diagnosis of time sensitive pathologies such as pneumothorax can enable earlier treatment, potentially reducing recovery time and the risk of complications. Moreover, reducing diagnostic errors can protect radiologists from medical malpractice claims.

Two previous studies evaluated AI software to identify missed findings in a selection of chest radiographs that were reported as normal [30, 31]. Hwang et al [30] found relevant missed abnormalities in 2.4% (103/4208) and false-positives in 14.0% (591/4208) of cases. Kaviani et al [31] reported a high number of clinically important missed findings of 11.3% (273/2407), as identified by radiologists. They applied a commercial AI tool to detect these missed findings, but only reported area under the curve (AUC) values which could not be compared to our study. Nevertheless, we found a lower percentage of clinically relevant missed findings (0.1%), most probably due to the broader inclusion criteria, using all available reports, and stricter definition of clinically relevance. We only deemed unreported findings relevant if they could alter the patient’s outcome. Another distinction with previous studies was our simultaneous AI-based analysis of imaging and reports. Such an automated comparative analysis is likely a requirement to clinically adopt an AI-assisted double reading system. Previous studies used a similar approach of combined imaging and report analysis to find relevant missed lung nodules on CT scans [32–34]. In a recent study by Cavallo et al [32], non-certified AI software was investigated to detect unreported lung nodules larger than 6 mm on emergency CT scans. In this specific setting, the tool flagged only 0.3% (50/19,246) of analysed CTs for discrepancies which, after review, resulted in 34 reports (68%) to receive an addendum. Our study was performed in a broader context on multiple pathologies and resulted in a higher number of AI-detected discrepancies (21.1%), requiring an external radiologist to review the results and assess their clinical relevance. When the AI-assisted double reading system is applied in a clinical setting, it is important that the external review of discrepancies is performed without significant delay to ensure that radiologists are notified on missed pathologies in a timely manner. The dedicated platform used in our study facilitated an efficient review process by presenting all cases in a convenient list. The radiologist categorised cases with potential discrepancies in a binary manner to allow a short review time. This approach is likely necessary for achieving cost-efficiency when applied in clinical practice. Future efforts should focus on reducing the number of flagged cases without clinical relevance to allow an automated double reading workflow, thus without needing assistance from an external radiologist. This will likely increase the cost-effectiveness of an AI-based double reading system.

Nevertheless, applying AI software as a second reader after report authorisation does not interrupt the primary workflow of the radiologist. Our results show that in the majority of radiographs (78.9%), AI analysis did not provide additional findings in comparison to the report. Therefore, the second reader approach avoids many unnecessary AI suggestions that, in a concurrent reader setting, would have no impact on the radiologist’s interpretation. Moreover, it reduces the risk of inaccurate AI suggestions to negatively influence the radiologist’s decision-making [35].

The study has limitations. First, we did not assess the standalone diagnostic performance of the investigated AI tool. The sole aim of the study was to assess the benefit of an AI-assisted double reading system. A previous multicentre study by Plesner et al evaluated the same AI algorithm which was configured to autonomously report normal chest radiographs and found it to have a higher sensitivity for abnormal chest radiographs (99.1%) than radiology reports (72.3%) [36]. Second, the double reading system focused mainly on identifying perceptual errors when the radiologist failed to detect and report the finding. It is by far the most common type of radiologic error (60–80%) [37, 38]. However, our approach might not identify some cognitive errors, in cases where the radiologist reported the finding but misinterpreted its significance. For example, a lung nodule or consolidation could have been described in the report and misclassified as benign. In the latter situation, the AI software would have not flagged the case for further review. Third, chest radiographs with AP projection were not eligible to be analysed by the regulatory-cleared AI software and were therefore excluded from this study. Fourth, a patient’s prior imaging examinations were not incorporated into the AI analysis and were not accessible to the external radiologist during review. Consequently, this restriction potentially hindered the ability of the external radiologist to detect any findings that may have been missed in follow-up examinations, particularly in cases where the report was less comprehensive or descriptive. Fifth, the clinical relevance of missed findings was determined by radiologists since data on patient outcomes were not available. Future studies should evaluate the AI-assisted double reading system prospectively in a clinical environment to assess if radiologists are notified about missed findings in a timely manner, and to assess the impact of missed findings on patient outcomes.

In conclusion, we demonstrated that an AI-assisted double reading system can identify unreported findings on chest radiographs. The occurrence of clinically relevant missed findings was very low in both institutions. The AI-assisted double reader approach could be applied as a quality assurance tool for the reporting of chest radiographs to mitigate diagnostic errors without interrupting the primary workflow of the radiologist.

### Supplementary Information

Below is the link to the electronic supplementary material.

Supplementary file1 (PDF 3382 KB)

## Abbreviations

AI: Artificial intelligence
AP: Anteroposterior
NLP: Natural language processing
PA: Posteroanterior

## Declarations

### Guarantor

The scientific guarantor of this publication is Erik R. Ranschaert.

### Statistics and biometry

One of the authors (Renee Menezes) has significant statistical expertise.

### Study subjects or cohorts overlap

No study subjects have been previously reported.

### Methodology

• retrospective

• observational study

• multicentre study
