# Supplementary material for: Artificial intelligence-assisted double reading of chest radiographs to detect clinically relevant missed findings: a two-centre evaluation
Source: Eur Radiol. 2024 Mar 11;34(9):5876–85. doi: 10.1007/s00330-024-10676-w (PMC11364654; doi:10.1007/s00330-024-10676-w)
Supplement: Supplementary file 1 — Supplementary file1 (PDF 3382 KB) [file 330_2024_10676_MOESM1_ESM.pdf]

Artificial intelligence-assisted double reading of chest radiographs to detect clinically relevant missed findings: a two-centre evaluation

ELECTRONIC SUPPLEMENTARY MATERIAL

Eur Radiol (2024) Topff L, Steltenpool S, Ranschaert ER, et al.

Supplementary Figure S1. A dedicated analytics platform was used to review chest radiographs and reports with artificial intelligence (AI)-detected discordant findings. The cases were displayed in a list view that allowed the reviewer to scroll through. The AI-detected findings were shown as a coloured overlay on the images.

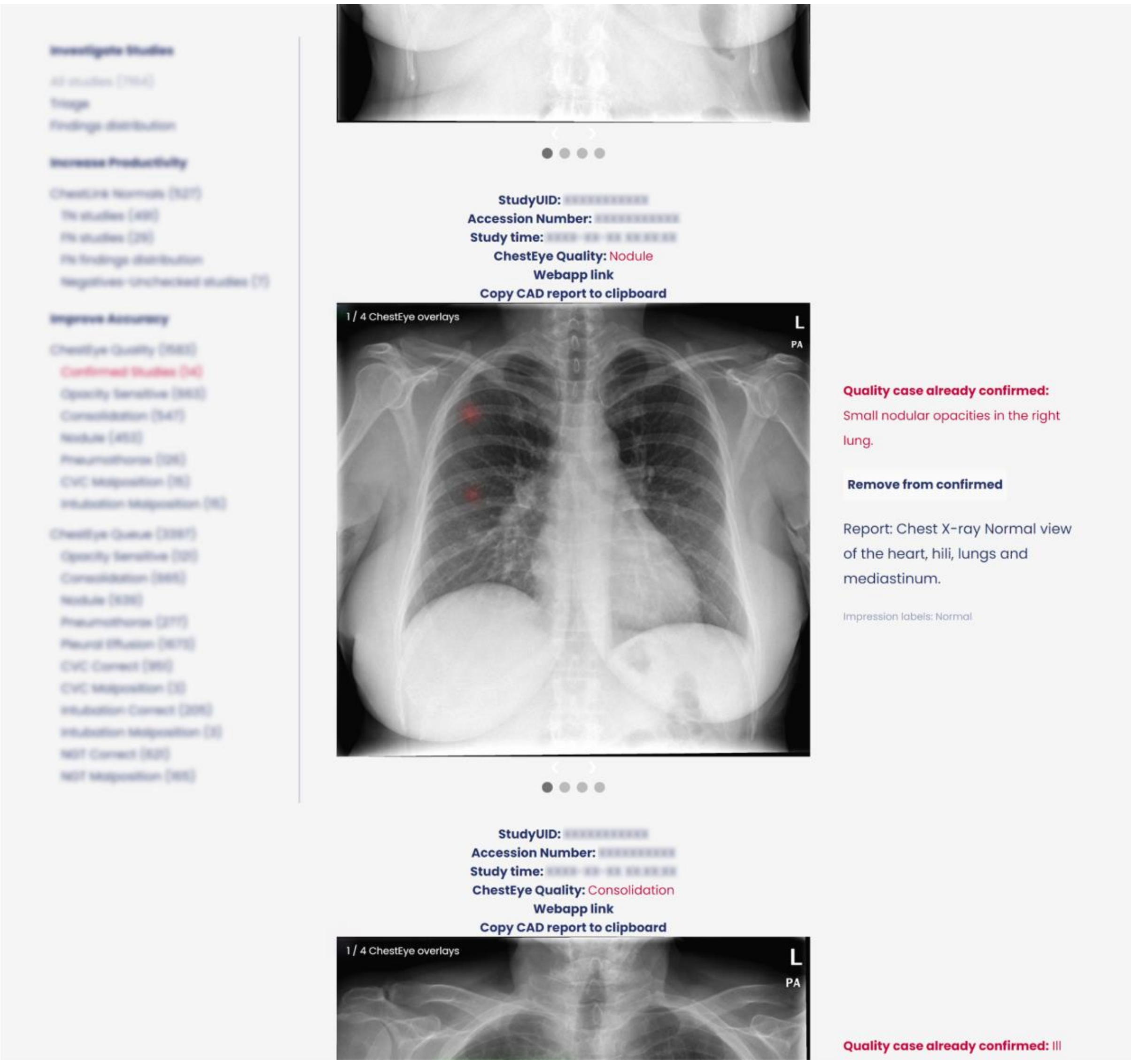

Appendix A. Detailed list of missed findings per institution

Institution 1: General hospital

Case 1

**Original chest radiograph**<br>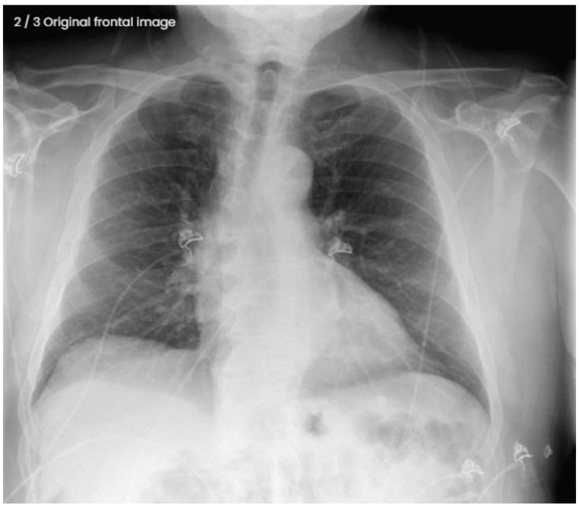	**Original radiology report (automatic translation)**<br>For comparison X-thorax of XX-XX-XXXX. Slim mediastinum superior. Hili well delimitable. Aperture domes sharply delimitable. Sinus pleurae well prosecute. No pleural fluid, no delineated infiltrates. No pulmonary nodular abnormalities. No thrust features. Normal heart size with sharp heart contour. Elongatio aortae. Degenerative abnormalities of the TWK. No osteolysis.<br><br>**NLP labels**<br>Normal
**Overlay of AI-detected findings (in color)**<br>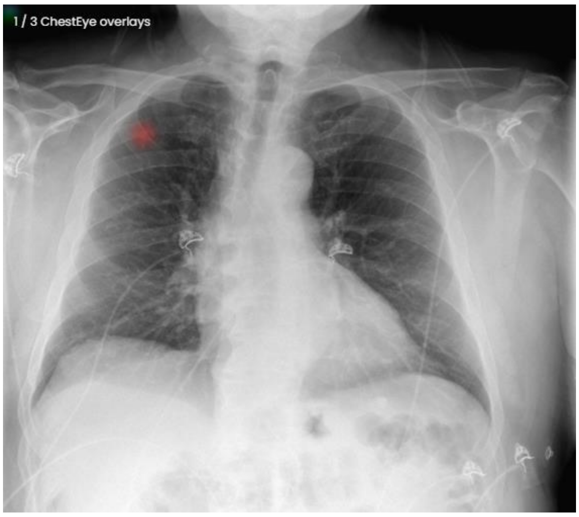	**AI-detected findings (ChestEye Quality)**<br>Opacity Sensitive
**External radiologist assessment**<br>Small right lung upper field nodular opacity.<br><br>**Institution's radiologist assessment**<br>Confirmed	

Case 2

Original chest radiograph

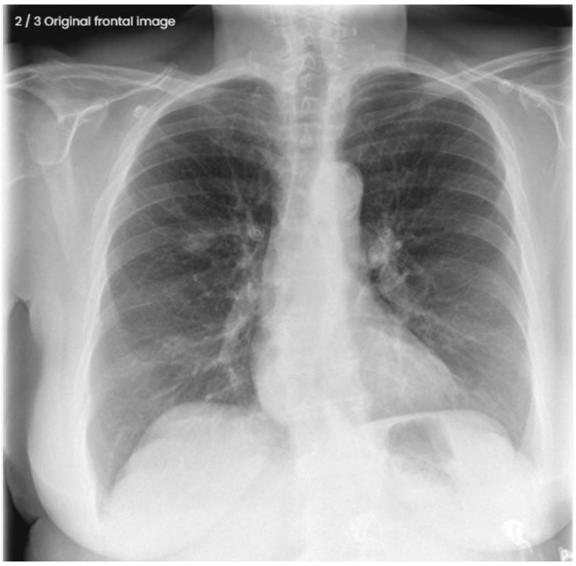

Original radiology report (automatic translation)

Compared to XX XXXX. Two-way research. Good inspiration stand. Sharply delimitable aperture domes with bright costofrenic sine wave. Normal cardiomediastinum. Normal hili and peripheral pulmonary vascular drawing. There are no compactions suitable for infiltrates. No pleural fluid. Known height loss of thoracic 11 and 7, with a severe kyphosis low thorcal. No new slumps.

NLP labels

Normal

Overlay of AI-detected findings (in color)

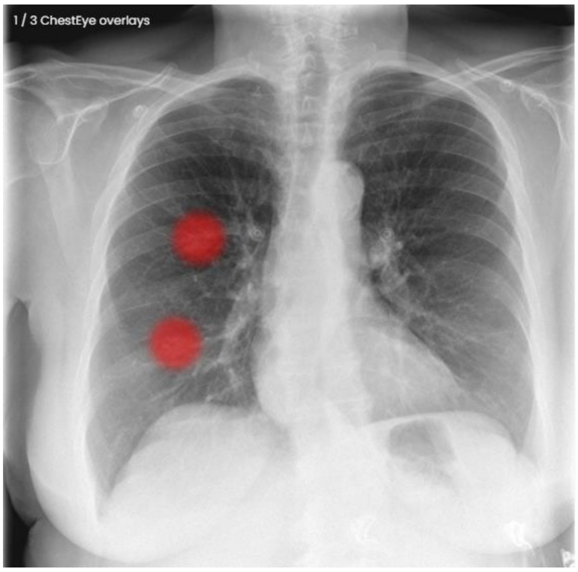

AI-detected findings (ChestEye Quality)

Opacity Sensitive

External radiologist assessment

Nodular opacity - *no additional comment available*

Institution's radiologist assessment

Confirmed

Case 3

Original chest radiograph

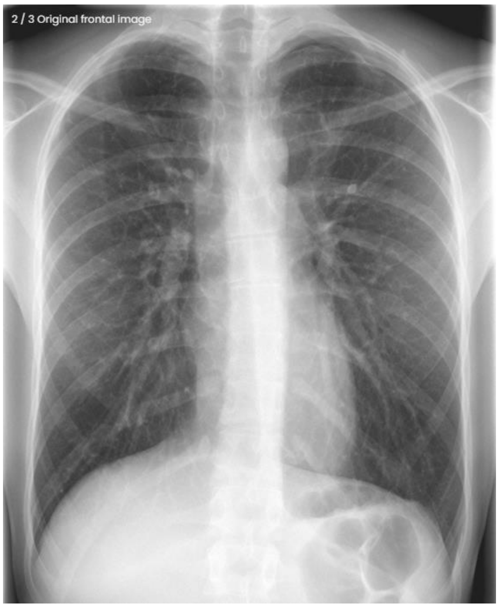

Original radiology report (automatic translation)

Compared to XX XX XXXX. Slim cor and mediastinum. Some busy drawing perihilar befitting bronchiids. No delimitable infiltrate. No pleural fluid.

NLP labels

Congestion, Follow_Up, Tracheal_Stent

Overlay of AI-detected findings*

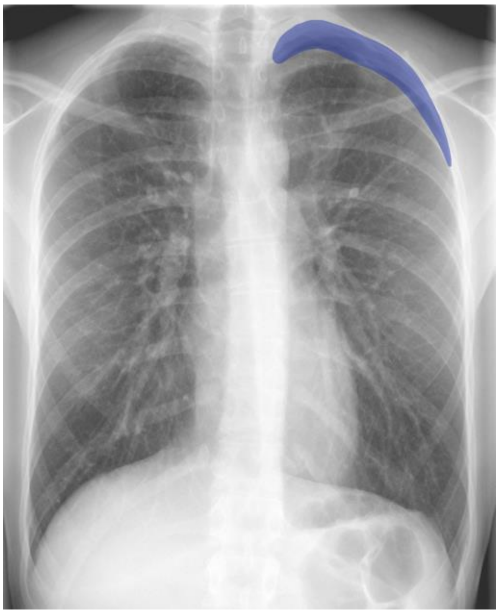

AI-detected findings (ChestEye Quality)

Pneumothorax

External radiologist assessment

Left apical lucency consistent with small apical pneumothorax.

Institution's radiologist assessment

Confirmed

Case 4

Original chest radiograph

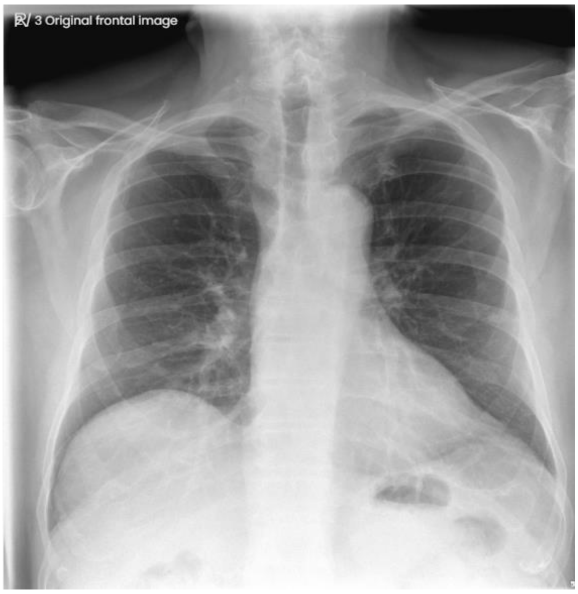

Original radiology report (automatic translation)

It has been compared with the survey of X XX, XXXX. The heart is normal in size. Slender mediastinum at normal hili. Both lungs show no abnormalities. The skeleton is intact. No changes since last survey.

NLP labels

Follow_Up

Overlay of AI-detected findings (in color)

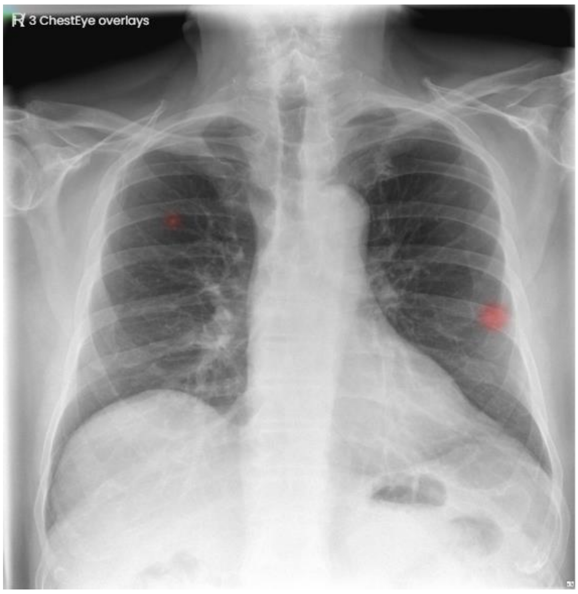

AI-detected findings (ChestEye Quality)

Opacity Sensitive

External radiologist assessment

Nodular opacity in the left lung.

Institution's radiologist assessment

Confirmed

Case 5

Original chest radiograph

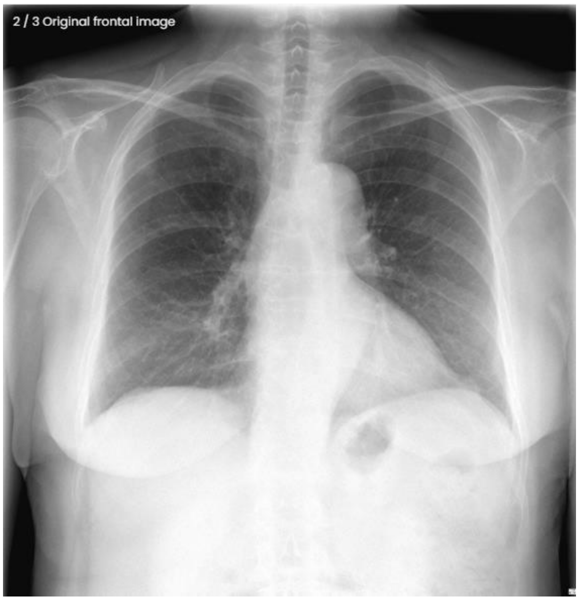

Original radiology report (automatic translation)
Heart and lungs no particulars. No infiltrates. Image in accordance with previous research from XXXX

NLP labels
Follow_Up

Overlay of AI-detected findings (in color)

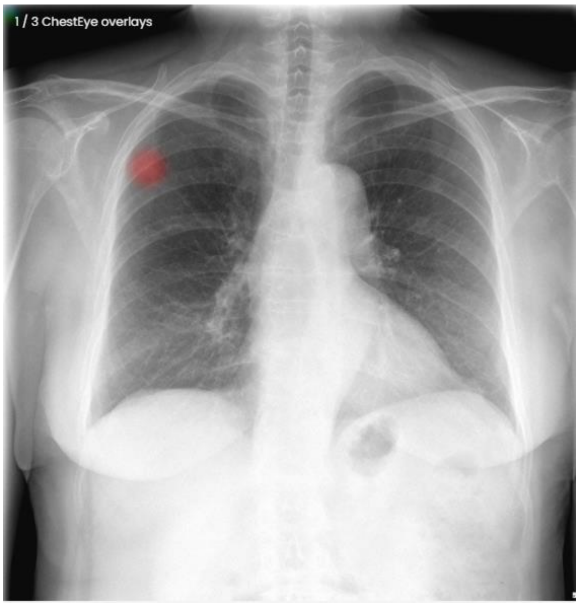

AI-detected findings (ChestEye Quality)
Opacity Sensitive

External radiologist assessment
Subtle possible nodular opacity in the upper right lung.

Institution's radiologist assessment
Confirmed

Case 6

<table>
<tr>
<td>

Original chest radiograph

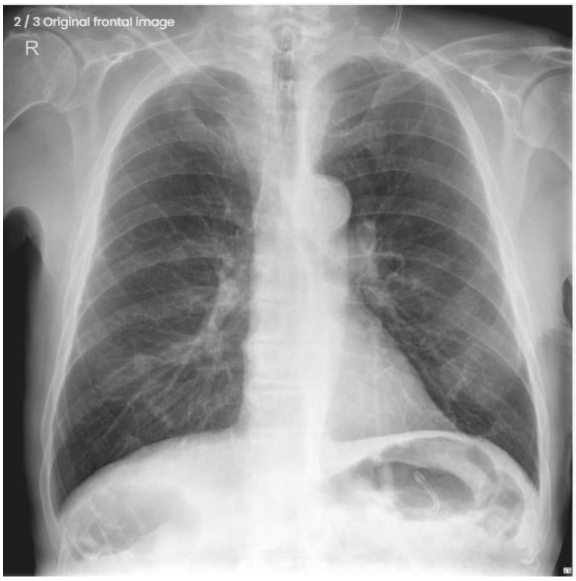

</td>
<td>

Original radiology report (automatic translation)
Normal cor size. Presence of gavage, good position. No infiltrates in the lungs. Mediastinum is not widened.

NLP labels
Nasogastric_Tube, Widened_Mediastinum

</td>
</tr>
<tr>
<td>

Overlay of AI-detected findings (in color)

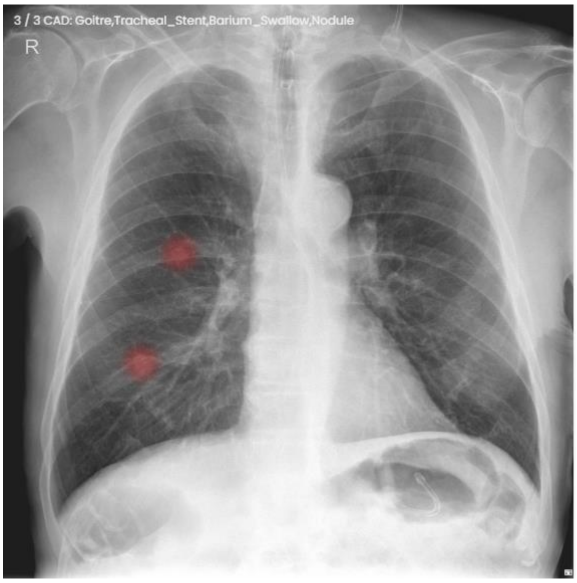

</td>
<td>

AI-detected findings (ChestEye Quality)
Nodule

</td>
</tr>
<tr>
<td colspan="2">

External radiologist assessment
Nodular opacity - *no additional comment available*

Institution's radiologist assessment
Confirmed

</td>
</tr>
</table>

Case 7

<table>
<tr>
<td>Original chest radiograph
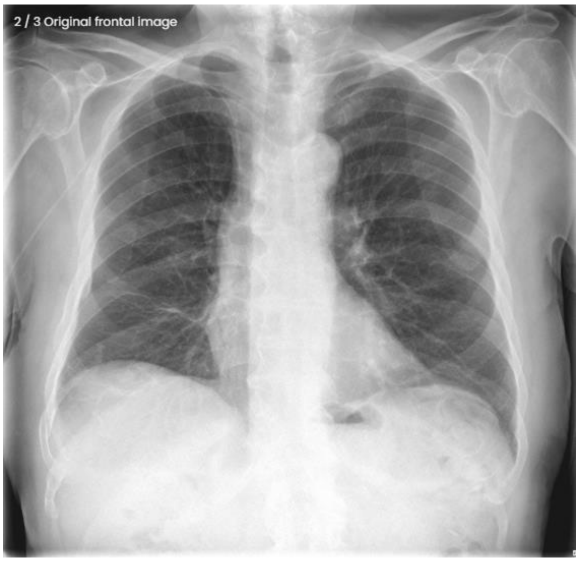</td>
<td>Original radiology report (automatic translation)
Heart and lungs gb degenerative brackets TWK

NLP labels
Normal</td>
</tr>
<tr>
<td>Overlay of AI-detected findings (in color)
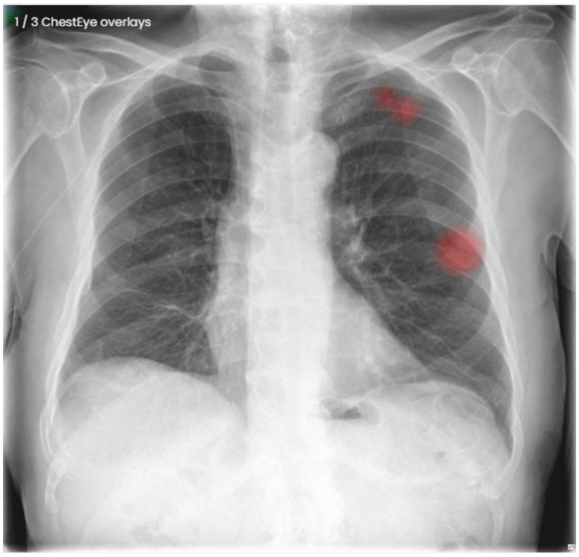</td>
<td>AI-detected findings (ChestEye Quality)
Opacity Sensitive</td>
</tr>
<tr>
<td colspan="2">External radiologist assessment
Small left lung upper and middle field nodular opacity.

Institution's radiologist assessment
Confirmed</td>
</tr>
</table>

Case 8

Original chest radiograph

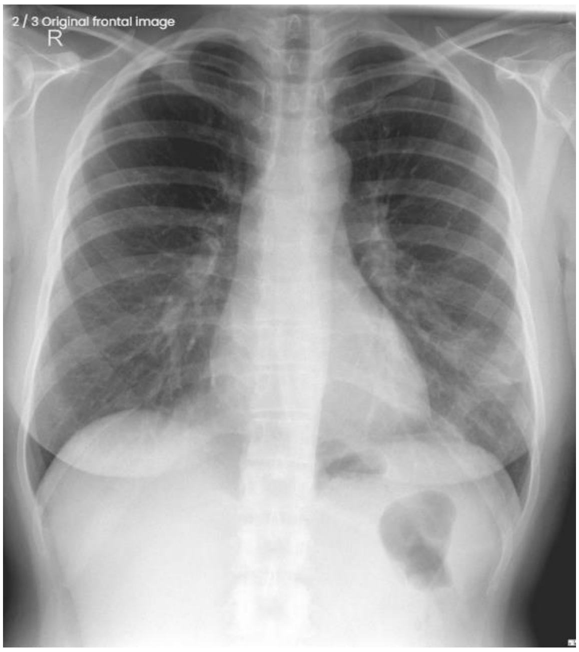

Original radiology report (automatic translation)

Good symmetrical inspiration stand. No soft tissue and skeletal abnormalities. Normally arched diaphragm domes and bright sine pleurae. The heart is not enlarged. To hili, mediastinum and lungs no particulars.

NLP labels

Normal

Overlay of AI-detected findings (in color)

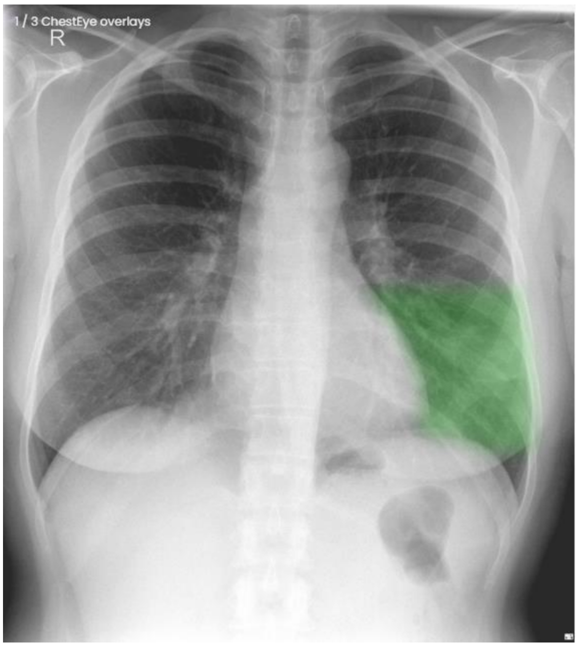

AI-detected findings (ChestEye Quality)

Consolidation

External radiologist assessment

Consolidation basal part of left lung.

Institution's radiologist assessment

Confirmed

Case 9

Original chest radiograph

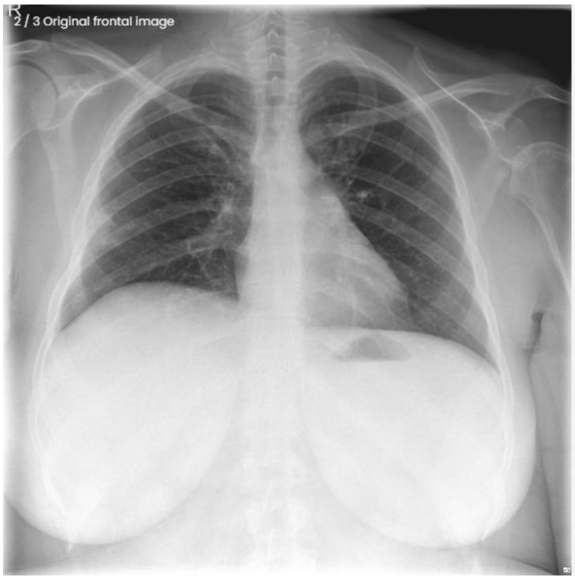

Original radiology report (automatic translation)
No abnormalities in the heart and lungs. No pleural fluid. As far as assess no fractures. No specifics to mediastinum hili.
Conclusion. No deviations.

NLP labels
Normal

Overlay of AI-detected findings (in color)

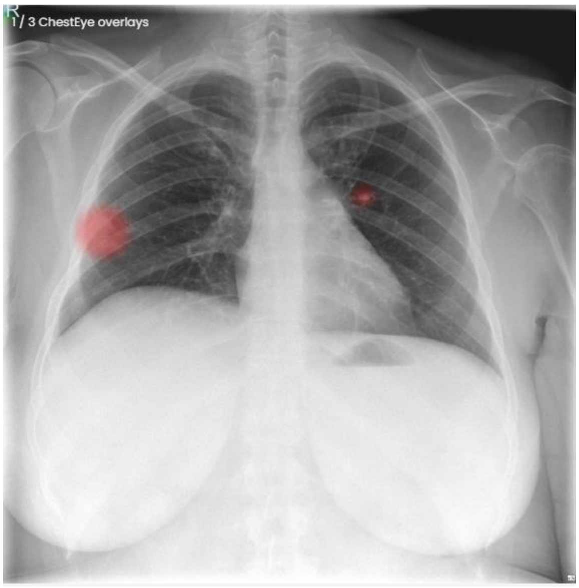

AI-detected findings (ChestEye Quality)
Opacity Sensitive

External radiologist assessment
Right lung middle field ill defined opacities.

Institution's radiologist assessment
Confirmed

Case 10

Original chest radiograph

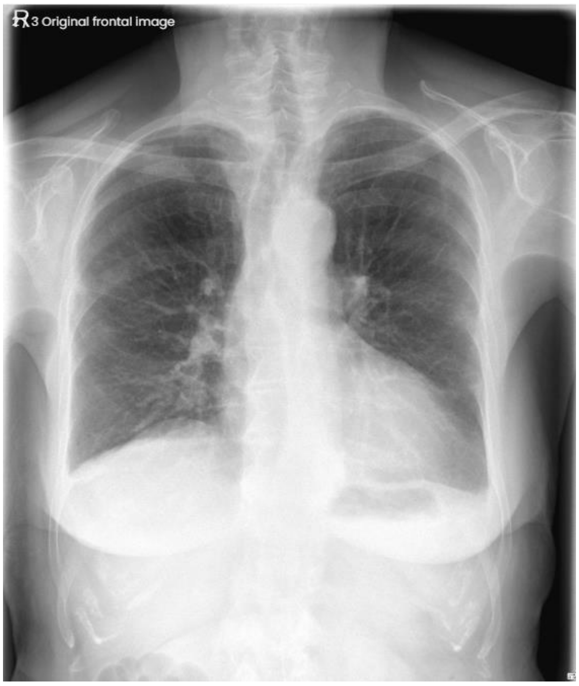

Original radiology report (automatic translation)

No previous research for comparison. Normal configuration of mediastinum. Normal cor size. Normal aspect of hili. No consolidations. No pleural fluid. Normal aspect of osseous structures and soft tissues. Conclusion: No evidence of TB or other abnormalities.

NLP labels

Normal

Overlay of AI-detected findings (in color)

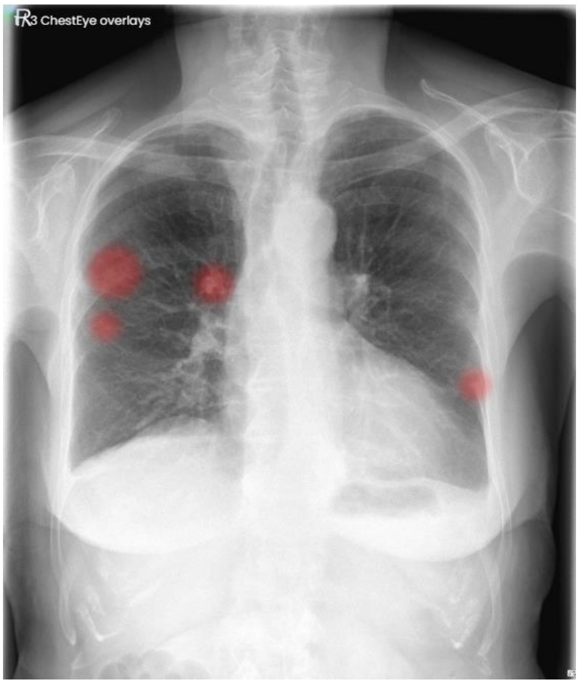

AI-detected findings (ChestEye Quality)

Consolidation, Opacity Sensitive

External radiologist assessment

Small densities are visible laterally on the right, at the axillary level.

Institution's radiologist assessment

Confirmed

Case 11

Original chest radiograph

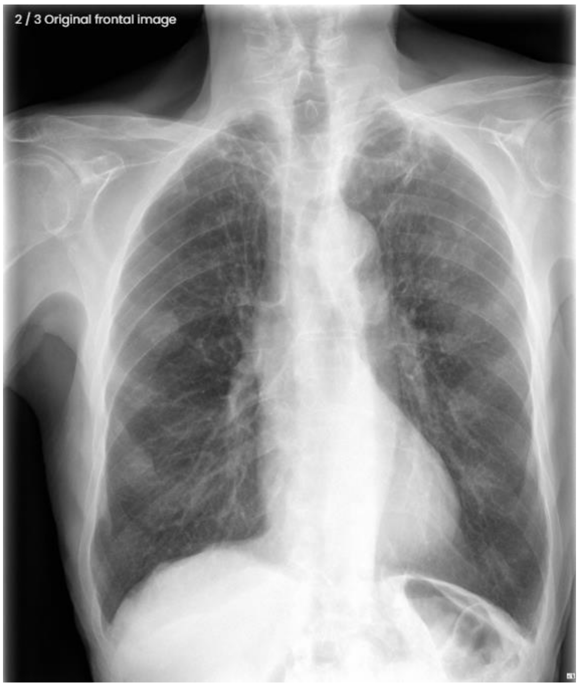

Original radiology report (automatic translation)

Two-way research. It is compared with the survey dated XX-XX-XXXX. Emphysematous thoracic image with bronchiectasis and bronchopathy. No indication of an added mass. Slim corcontour. Normal contour of mediastinum superior. Degenerative changes of depicted skeleton. No pneumothorax.

NLP labels

Bronchiectasis, Pulmonary_Emphysema

Overlay of AI-detected findings (in color)

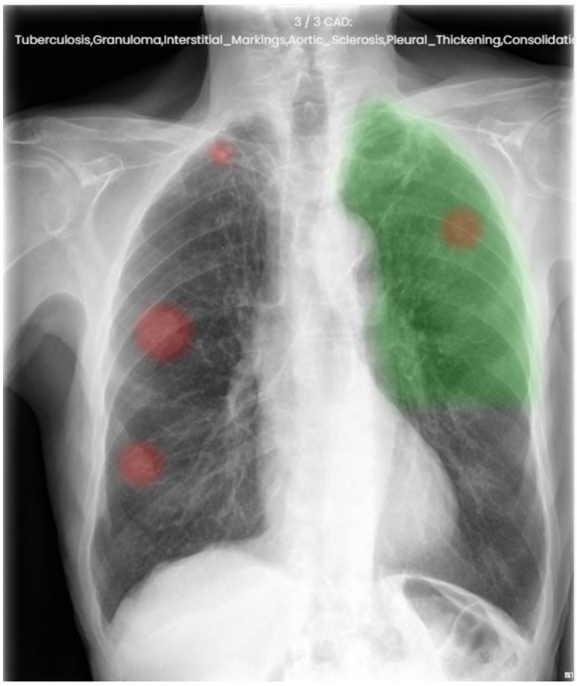

AI-detected findings (ChestEye Quality)

Consolidation, Nodule

External radiologist assessment

Nodular opacity - *no additional comment available*

Institution's radiologist assessment

Confirmed

Case 12

**Original chest radiograph**<br>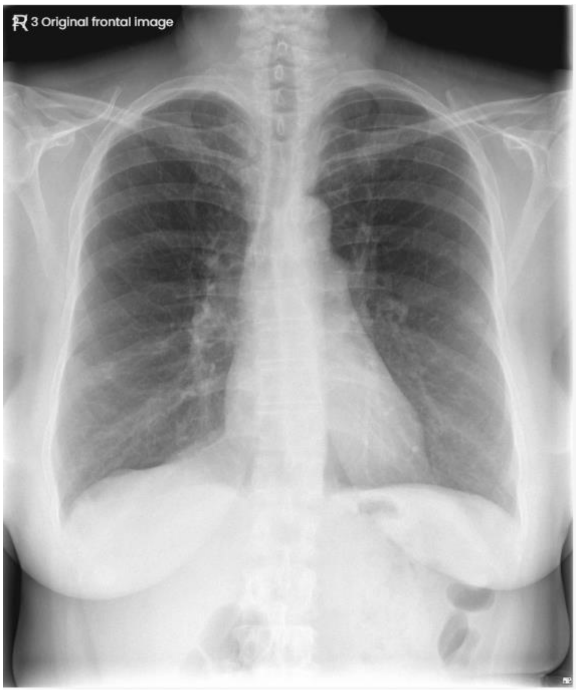 	**Original radiology report (automatic translation)**<br>Heart and lungs no particulars.<br><br>**NLP labels**<br>Normal
**Overlay of AI-detected findings (in color)**<br>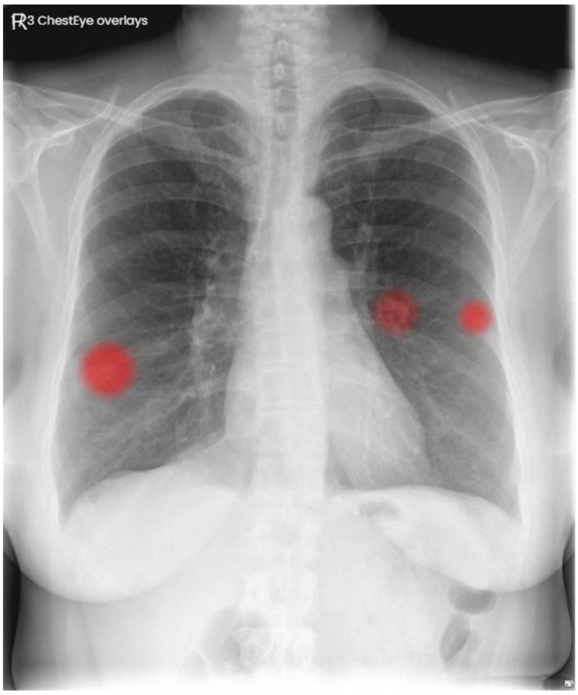 	**AI-detected findings (ChestEye Quality)**<br>Opacity Sensitive
**External radiologist assessment**<br>Nodular opacity - *no additional comment available*<br><br>**Institution's radiologist assessment**<br>Confirmed	

Case 13

Original chest radiograph

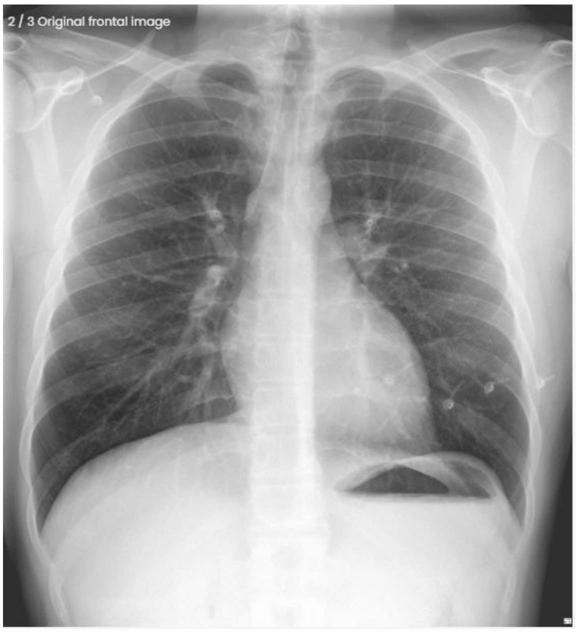

Original radiology report (automatic translation)

Two-way research. First research here. Sharply delimitable diaphragm domes sinus pleurae, no pleural fluid. Slim cardiomediastinum. Here pulmonary vascular drawing within the norm. Normal air retention of lung fields, no additional infiltrate. No pneumothorax. Intact thoracic skeleton.

NLP labels

Normal

Overlay of AI-detected findings (in color)

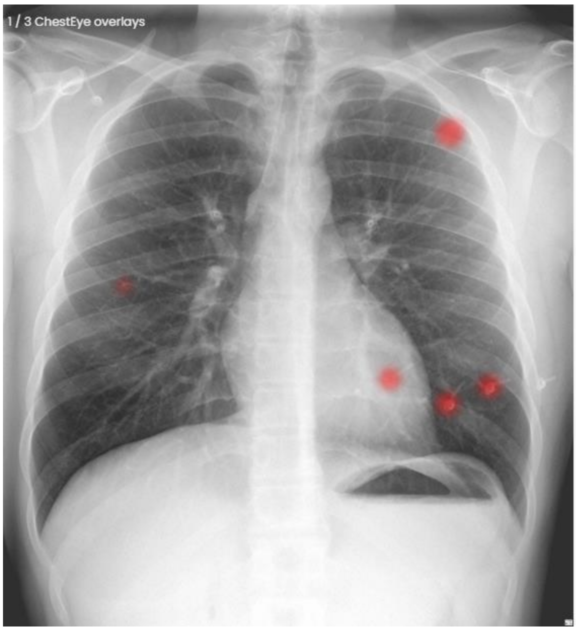

AI-detected findings (ChestEye Quality)

Opacity Sensitive

External radiologist assessment

Nodular opacity - *no additional comment available*

Institution's radiologist assessment

Confirmed

Case 14

<table>
<tr>
<td>

Original chest radiograph

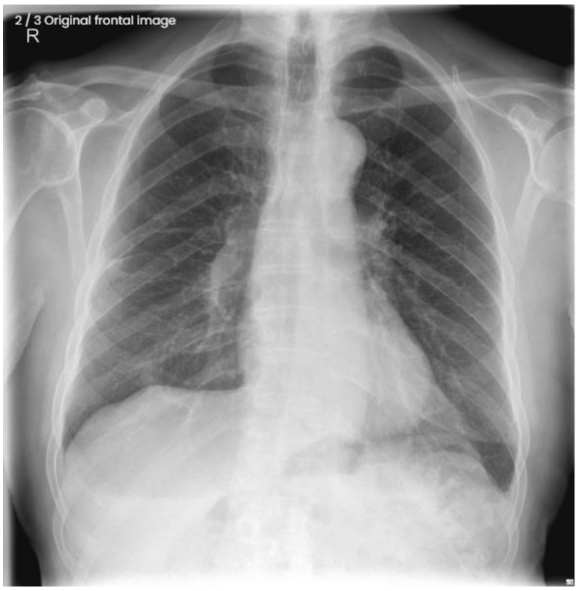

</td>
<td>

Original radiology report (automatic translation)
No old research. Well-inspired absorption, no active pathology to the heart or lungs. Normal image of hili and upper mediastinum.

NLP labels
Normal

</td>
</tr>
<tr>
<td>

Overlay of AI-detected findings (in color)

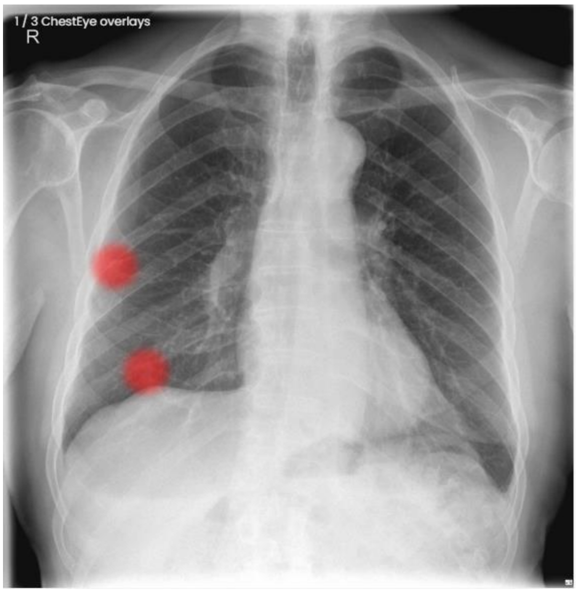

</td>
<td>

AI-detected findings (ChestEye Quality)
Opacity Sensitive

</td>
</tr>
<tr>
<td colspan="2">

External radiologist assessment
Nodular opacity - *no additional comment available*

Institution's radiologist assessment
Confirmed

</td>
</tr>
</table>

Case 15

Original chest radiograph

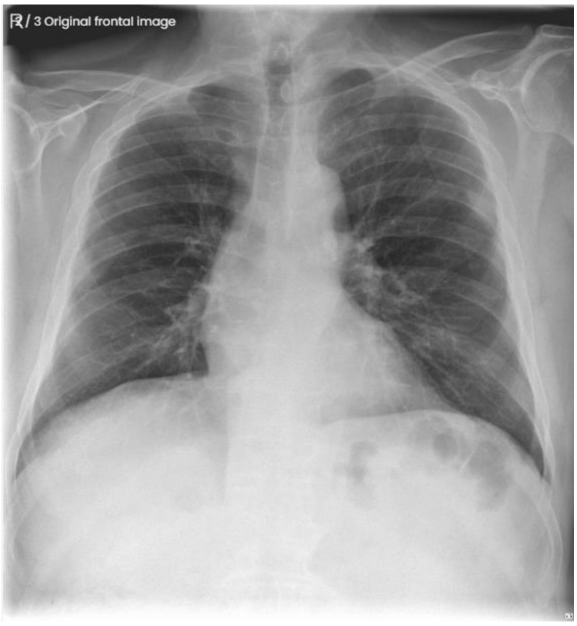

Original radiology report (automatic translation)

Standing chest X-ray in 2 directions. For comparison X-thorax of xx-xx-xxxx. Slender corfigure and mediastinum superior. Elongated thoracic aorta. Normal pulmonary vascular drawing. Volumen pulmonum auctum. No consolidations. No pleural fluid. No particulars to the skeleton.

NLP labels

Normal

Overlay of AI-detected findings (in color)

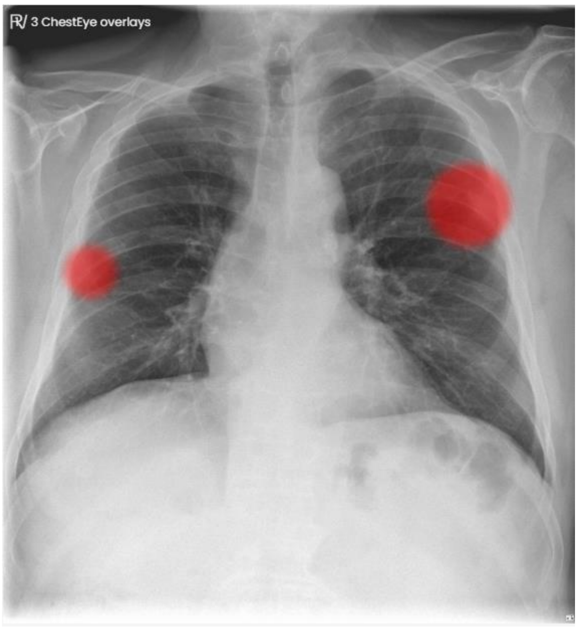

AI-detected findings (ChestEye Quality)

Opacity Sensitive

External radiologist assessment

Nodular opacity - *no additional comment available*

Institution's radiologist assessment

Confirmed

Case 16

Original chest radiograph

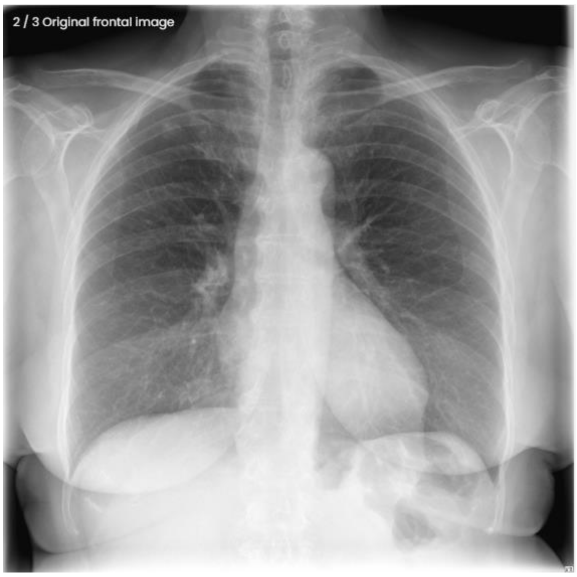

Original radiology report (automatic translation)
Compared to the survey of XX-XX-XXXX. PA and lateral recording. Good inspiration. Slim mediastinum and cor. The diaphragm domes are well delimitable with bright sine pleurae. No pleural abnormalities. Normal hili on both sides. No consolidations or nodular abnormalities. No soft tissue and skeletal abnormalities.

NLP labels
Normal

Overlay of AI-detected findings (in color)

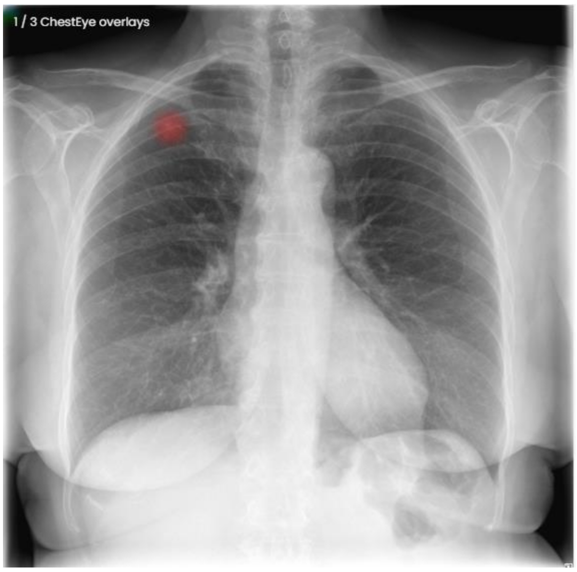

AI-detected findings (ChestEye Quality)
Opacity Sensitive

External radiologist assessment
Right lung upper field opacity.

Institution's radiologist assessment
Confirmed

Case 17

Original chest radiograph

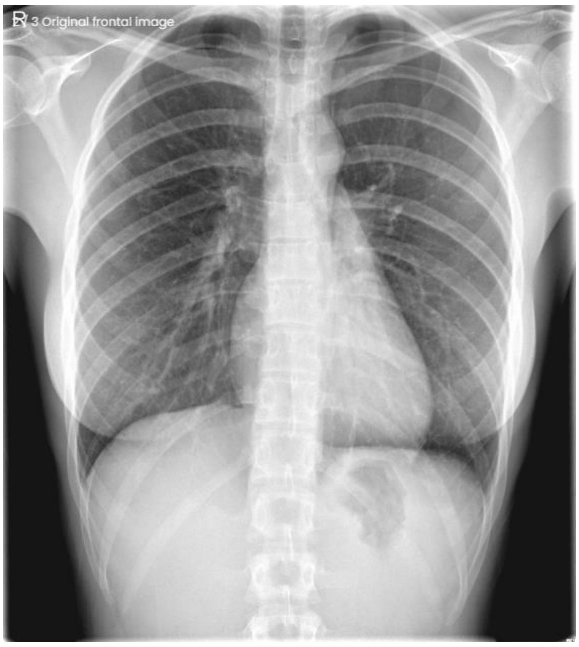

Original radiology report (automatic translation)

For comparison, the study of XX-XX-XXXX. PA and lateral recording. Good is inspirational stand. Slim cardiomediastinum. Pulmonary vascular drawing within the norm. Sharply delimitable aperture domes on both sides. No pleural fluid. No atelectasis. No circumscript compactions. Indication of malignancy. No pneumothorax. As far as to assess intact osseous structures.

NLP labels

Normal

Overlay of AI-detected findings*

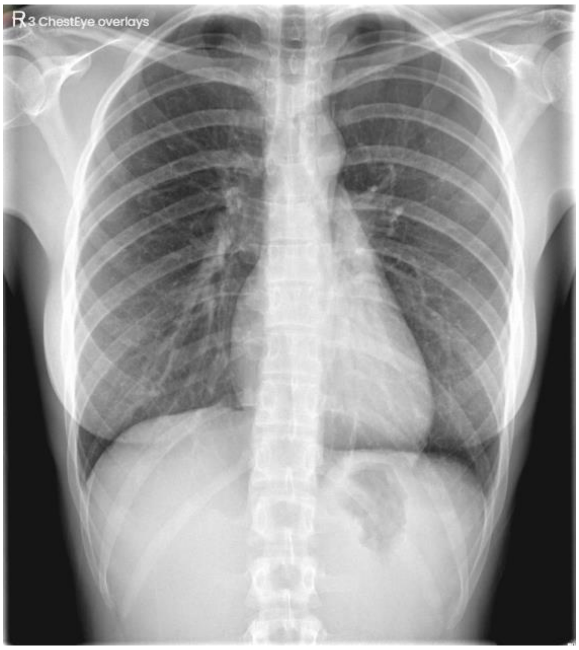

**Note: overlay was not available due to technical error*

AI-detected findings (ChestEye Quality)

Pneumothorax

External radiologist assessment

Left sided pneumothorax.

Institution's radiologist assessment

Confirmed

Case 18

Original chest radiograph

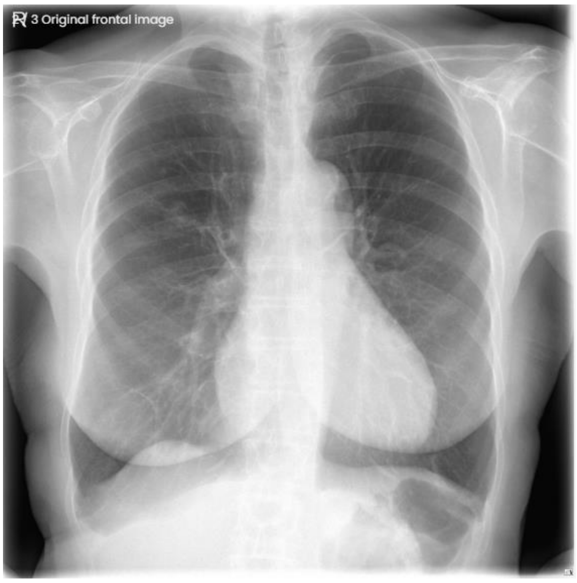

Original radiology report (automatic translation)

For comparison, XX-XX-XXXX. 2-way shooting. Heart size within the norm, slender mediastinum superius. Enlarged retrosternal space, spindly pulmonary vascular drawing and flattened diaphragm domes, matching emphysema. No suspected intrapulmonary lesions. No engorgement or infiltrates.

NLP labels

Retrosternal_Airspace_Obliteration, Pulmonary_Emphysema

Overlay of AI-detected findings (in color)

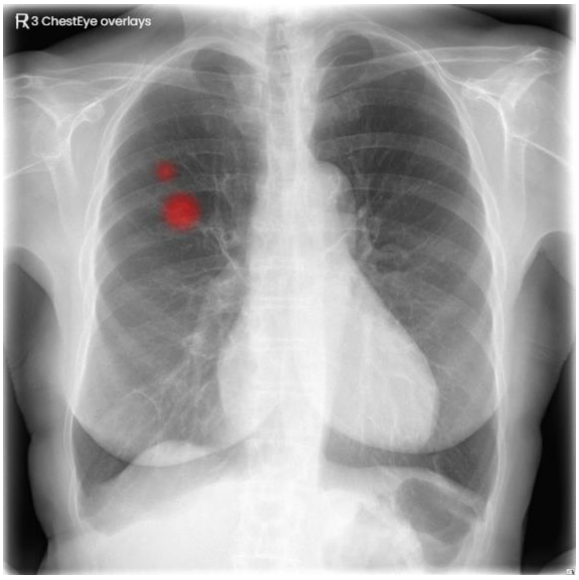

AI-detected findings (ChestEye Quality)

Opacity Sensitive

External radiologist assessment

Nodular opacity - *no additional comment available*

Institution's radiologist assessment

Confirmed

Case 19

<table>
<tr><td>

Original chest radiograph

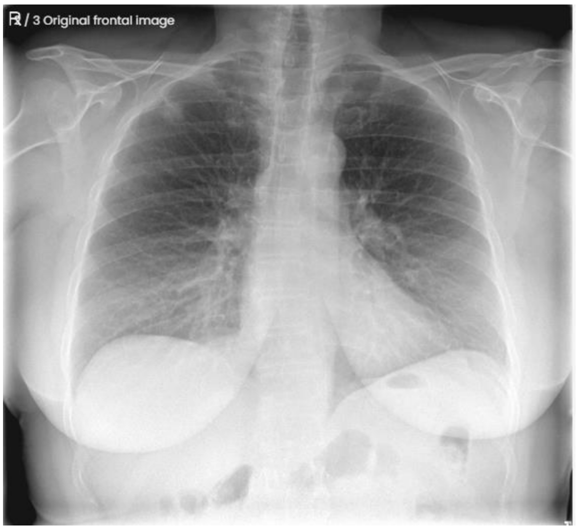

</td><td>

Original radiology report (automatic translation)

It is compared to XXXX; well-inspired admission without active pathology to the heart or lungs.

NLP labels

Normal

</td></tr>
<tr><td>

Overlay of AI-detected findings (in color)

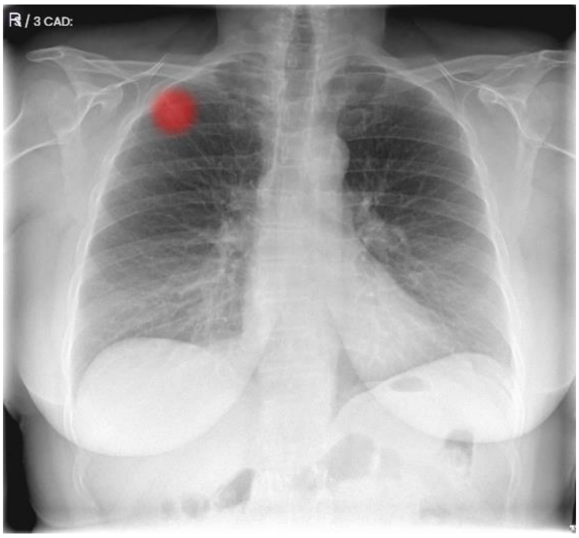

</td><td>

AI-detected findings (ChestEye Quality)

Consolidation, Opacity Sensitive, Nodule

</td></tr>
<tr><td colspan="2">

External radiologist assessment

Right lung upper field opacity, associated with mild pleural thickening.

Institution's radiologist assessment

Confirmed

</td></tr>
</table>

Case 20

Original chest radiograph

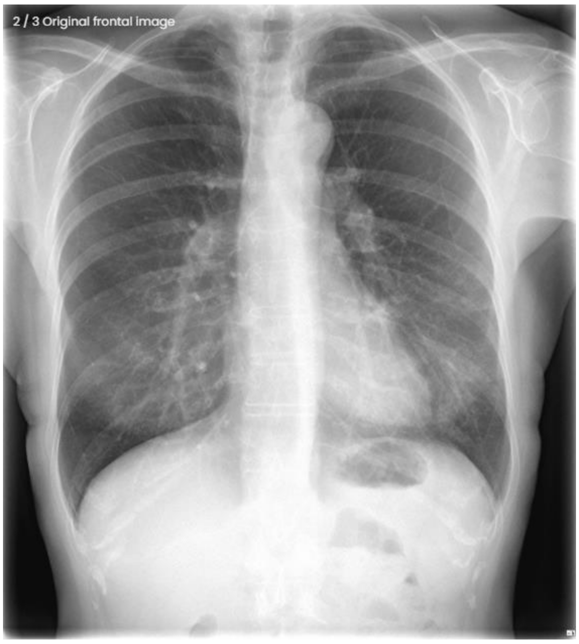

Original radiology report (automatic translation)
Compared to chest X-ray from XX-XX-XXXX. PA and lateral recording. Good inspiration. Slim mediastinum and cor. Aperture domes are easily delimitable. No pleural fluid. Normal hili on both sides. No consolidations or nodular abnormalities. No skeletal and soft tissue abnormalities.

NLP labels
Normal

Overlay of AI-detected findings (in color)

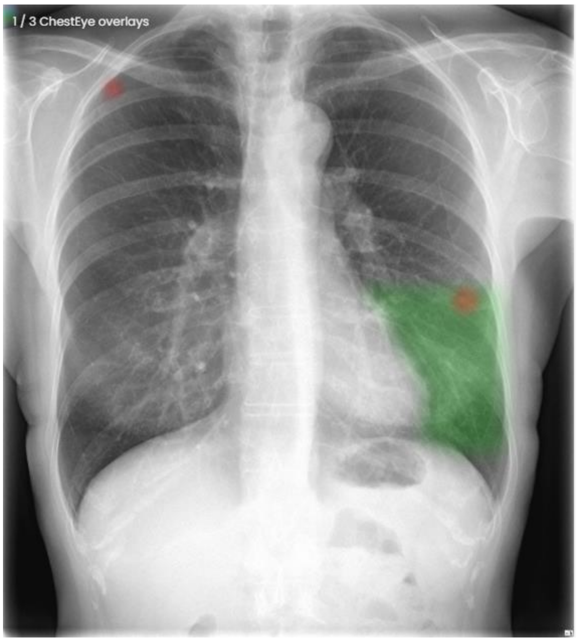

AI-detected findings (ChestEye Quality)
Consolidation, Opacity Sensitive.

External radiologist assessment
Ill defined opacities in the left lung middle field.

Institution's radiologist assessment
Confirmed

Case 21

<table>
<tr>
<td>

Original chest radiograph

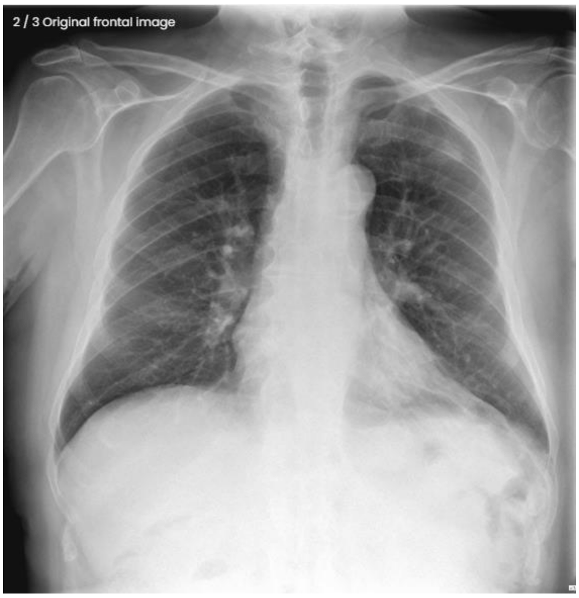

</td>
<td>

Original radiology report (automatic translation)
Two-way research. Status after previous lung puncture today. No pneumothorax or hematothorax. No extensive hematoma.

NLP labels
Normal

</td>
</tr>
<tr>
<td>

Overlay of AI-detected findings (in color)

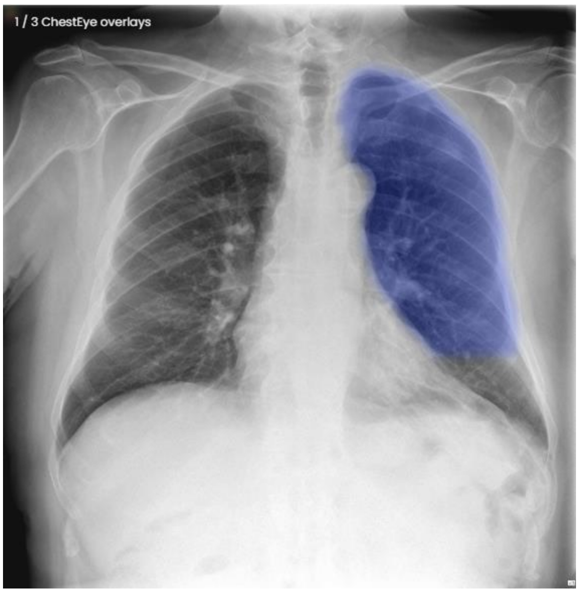

</td>
<td>

AI-detected findings (ChestEye Quality)
Pneumothorax

</td>
</tr>
<tr>
<td colspan="2">

External radiologist assessment
Pneumothorax - *no additional comment available*

Institution's radiologist assessment
Confirmed

</td>
</tr>
</table>

Case 22

Original chest radiograph

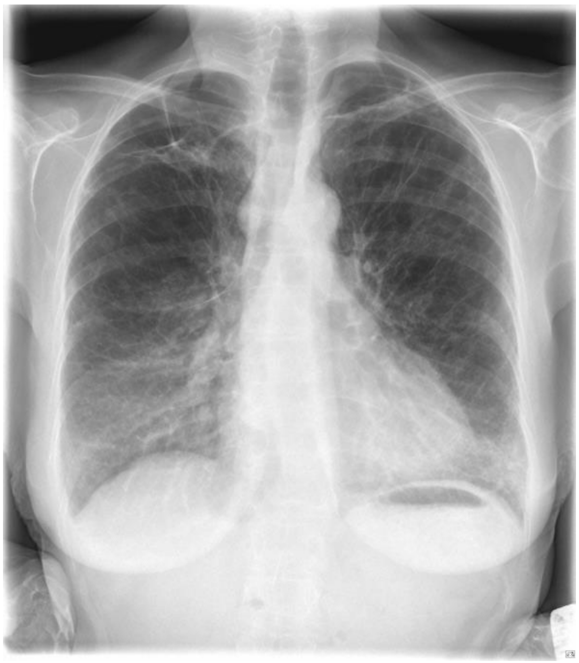

Original radiology report (automatic translation)

Compared to XX-XX-XXXX. Pre-existent COPD and emphysema. Bullae on both lung buds. Residual deviations at the location of the lower fields, on the left more than on the right.

NLP labels

Residual_Changes, Follow_Up, Bullous_Emphysema, Pulmonary_Emphysema

Overlay of AI-detected findings (in color)

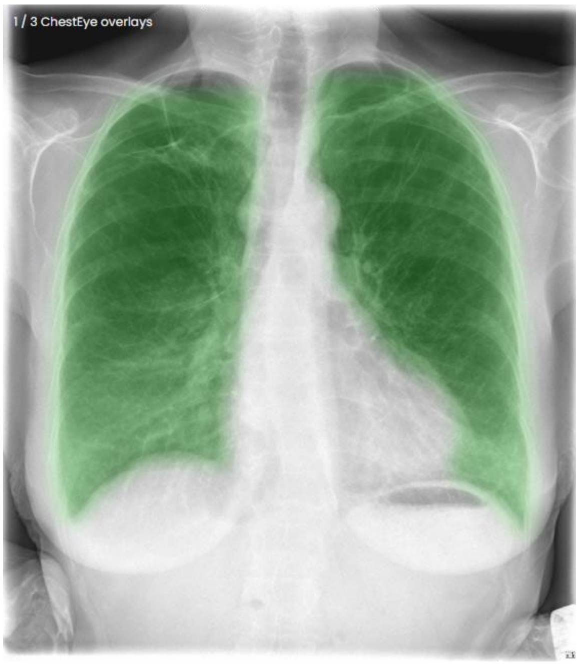

AI-detected findings (ChestEye Quality)

Consolidation

External radiologist assessment

Consolidation - *no additional comment available*

Institution's radiologist assessment

Rejected - finding was known and unchanged in comparison to prior imaging

Case 23

Original chest radiograph

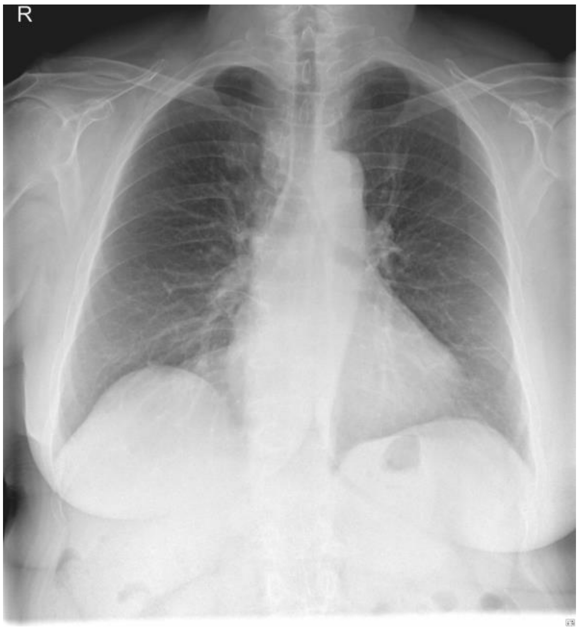

Original radiology report (automatic translation)

There are no thoracic vertebral collapses. An ankylosis of the thoracic spine has occurred. All bow feet are intact. Normal heart size. No abnormalities are visible in the heart and lungs.

NLP labels

Normal

Overlay of AI-detected findings (in color)

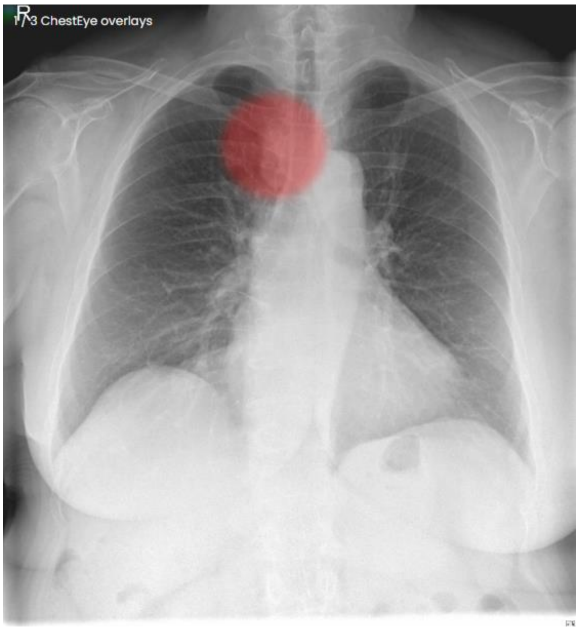

AI-detected findings (ChestEye Quality)

Opacity Sensitive

External radiologist assessment

Nodular opacity - *no additional comment available*

Institution's radiologist assessment

Rejected - finding was not unequivocally present

Case 24

Original chest radiograph

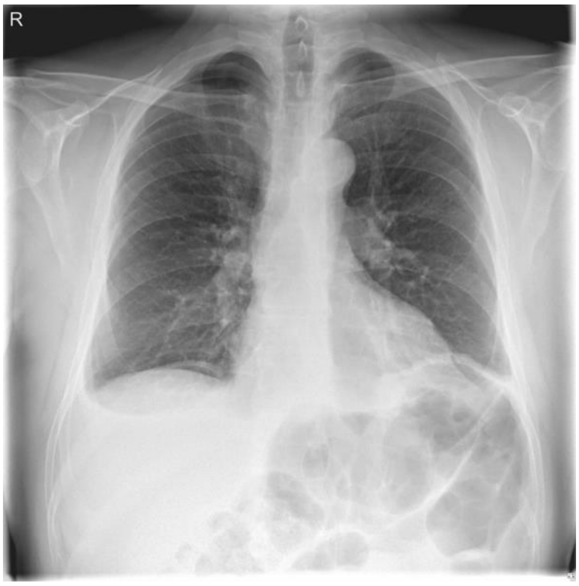

Original radiology report (automatic translation)

No previous research for comparison. After intra thoracic gastric reduction. Bilateral trace pleural fluid. Not an obvious pulmonary edema picture. No circumscript infiltrates. Slim cardiomediastinum.

NLP labels

Pleural_Effusion

Overlay of AI-detected findings (in color)

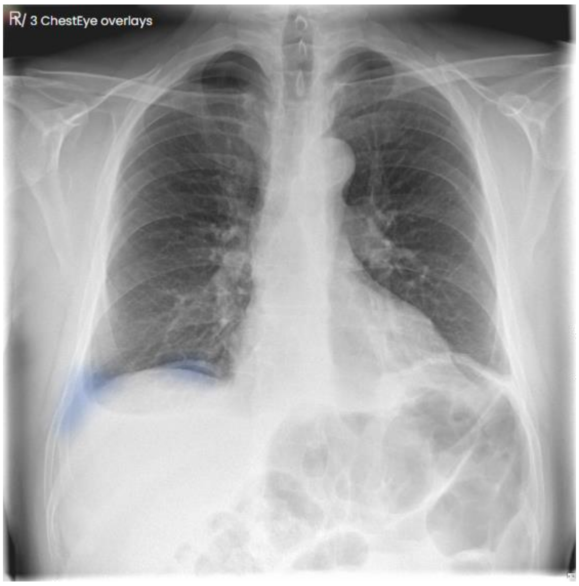

AI-detected findings (ChestEye Quality)

Pleural Effusion, Pneumothorax

External radiologist assessment

Pneumothorax - *no additional comment available*

Institution's radiologist assessment

Rejected - finding was not unequivocally present

Case 25

Original chest radiograph

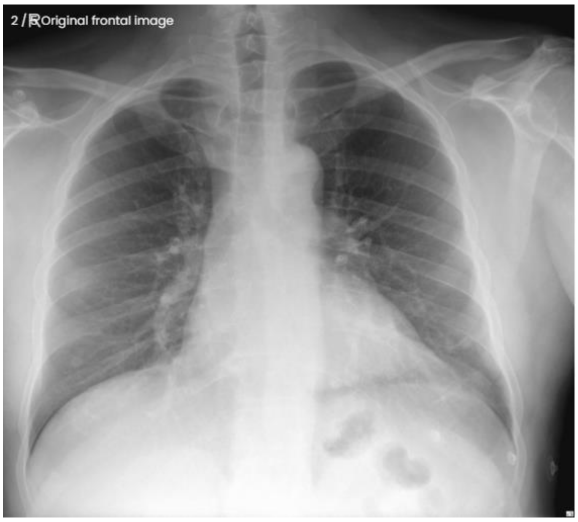

Original radiology report (automatic translation)

No research for comparison.Trachea in the midline. Sharply delimitable, symmetrical aperture domes. No pneumothorax or pleural fluid. Clear lung fields. Slender cor mediastinum superius. No thrust signs. No obvious rib fracture, no subcutaneous emphysema.

NLP labels

Normal

Overlay of AI-detected findings (in color)

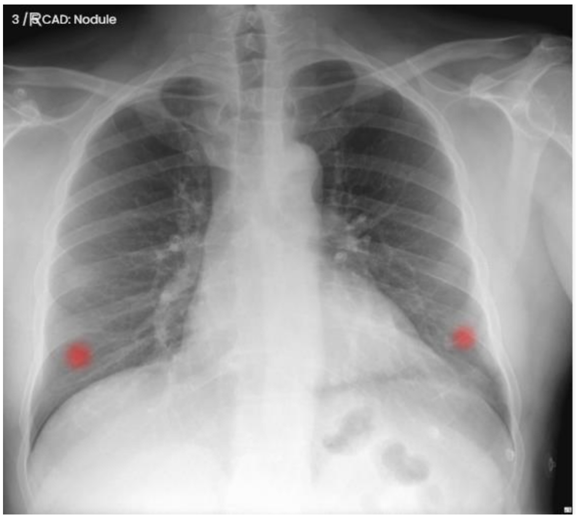

AI-detected findings (ChestEye Quality)

Opacity Sensitive, Nodule

External radiologist assessment

Nodular opacity - *no additional comment available*

Institution's radiologist assessment

Rejected - finding was not unequivocally present

Case 26

<table>
<tr>
<td>Original chest radiograph
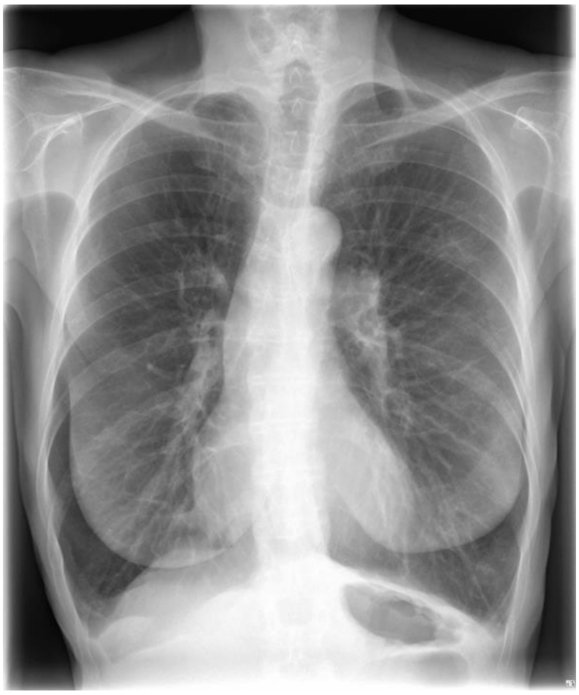</td>
<td>Original radiology report (automatic translation)
For comparison XX-XX-XXXX. Slim cor. No evidence of lymphadenopathy. Normal pulmonary vascular drawing. Volumen pulmonum auctum Clear lung fields. No pleural fluid. Minor right convex thoracic scoliosis.

NLP labels
Normal</td>
</tr>
<tr>
<td>Overlay of AI-detected findings (in color)
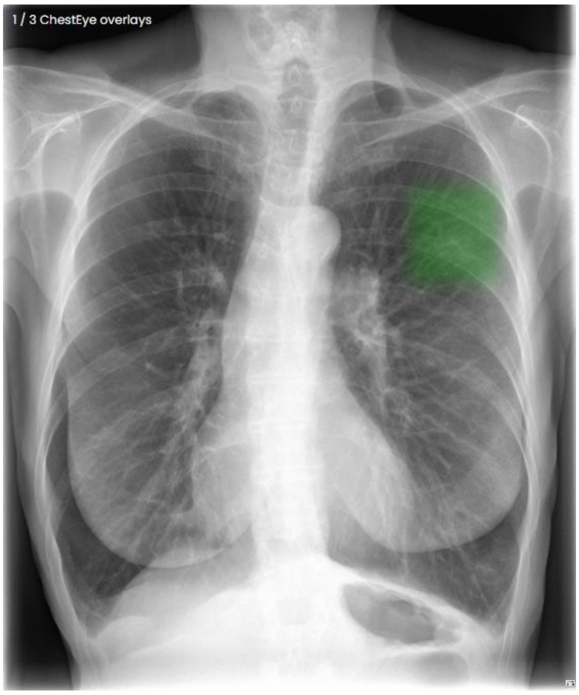
</td>
<td>AI-detected findings (ChestEye Quality)
Consolidation</td>
</tr>
<tr>
<td colspan="2">External radiologist assessment
Consolidation - no additional comment available

Institution's radiologist assessment
Rejected - finding was known and unchanged in comparison to prior imaging</td>
</tr>
</table>

Case 27

<table>
<tr>
<td>

Original chest radiograph

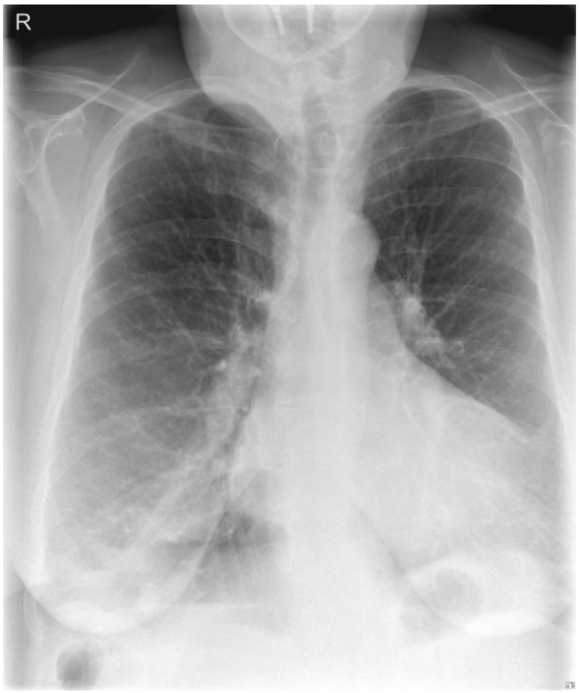

</td>
<td>

Original radiology report (automatic translation)

For comparison examination of XX-XX-XXXX. Known emphysematous chest image. Cardiomegaly. Pulmonary vascular drawing within the norm. No indication of lymphadenopathy. No pleural fluid. No consolidations. No indication of malignancy. Intact osseous structures.

NLP labels

Normal

</td>
</tr>
<tr>
<td>

Overlay of AI-detected findings (in color)

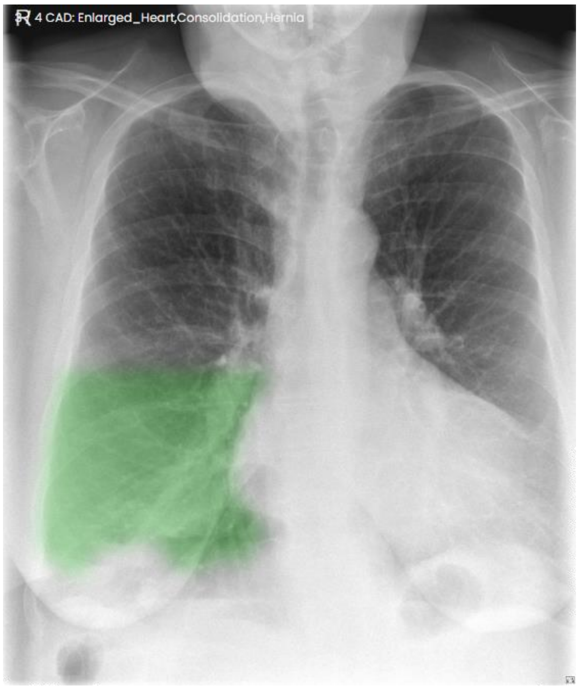

</td>
<td>

AI-detected findings (ChestEye Quality)

Consolidation

</td>
</tr>
<tr>
<td colspan="2">

External radiologist assessment

Consolidation - *no additional comment available*

Institution's radiologist assessment

Rejected - finding was not unequivocally present

</td>
</tr>
</table>

Case 28

Original chest radiograph

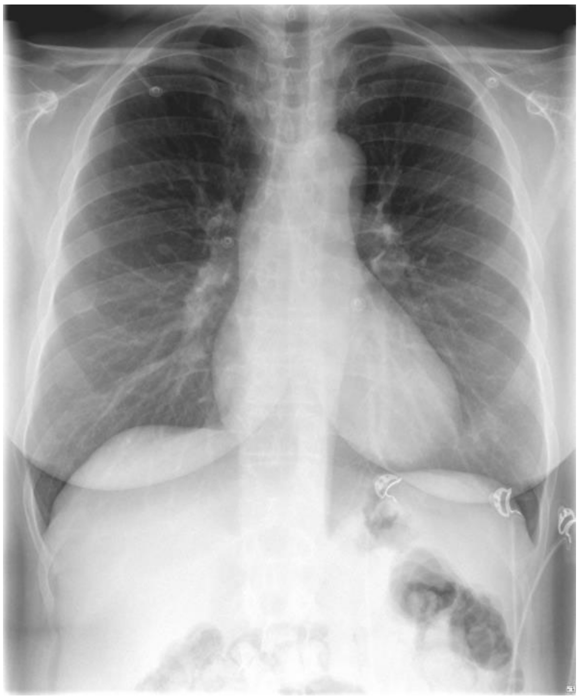

Original radiology report (automatic translation)
No soft tissue and skeletal abnormalities. Normally arched diaphragm domes and bright sine pleurae. The heart is not enlarged. To hili, mediastinum and lungs no particulars.

NLP labels
Normal

Overlay of AI-detected findings (in color)

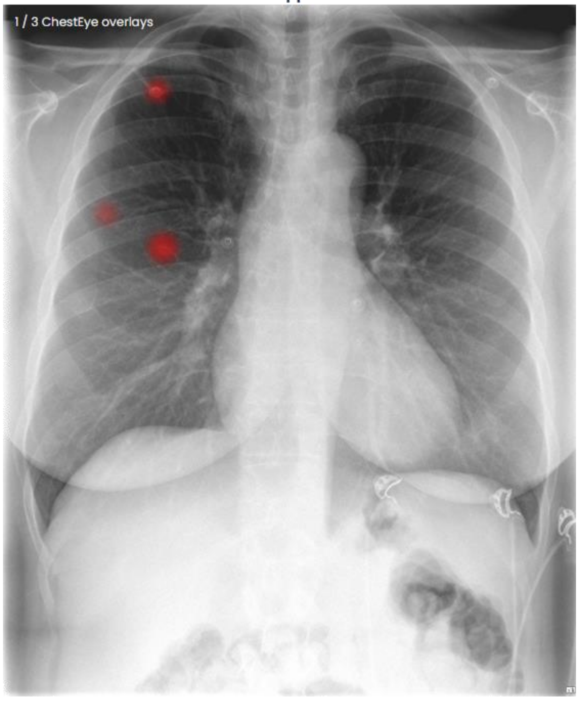

AI-detected findings (ChestEye Quality)
Opacity Sensitive

External radiologist assessment
Nodular opacity - *no additional comment available*

Institution's radiologist assessment
Rejected - finding was present but deemed not clinically relevant

Institution 2: Oncology centre

Case 1

<table>
<tr>
<td>Original chest radiograph
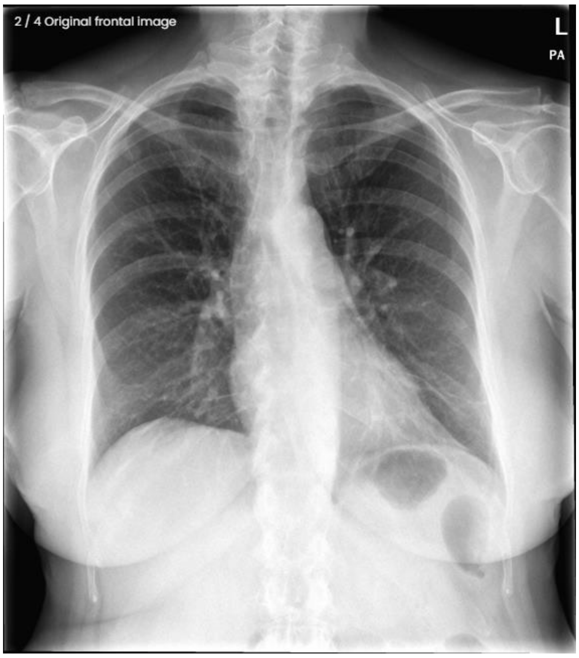
</td>
<td>Original radiology report (automatic translation)
Normal view of the heart, hili, lungs and mediastinum.

NLP labels
Normal</td>
</tr>
<tr>
<td>Overlay of AI-detected findings (in color)
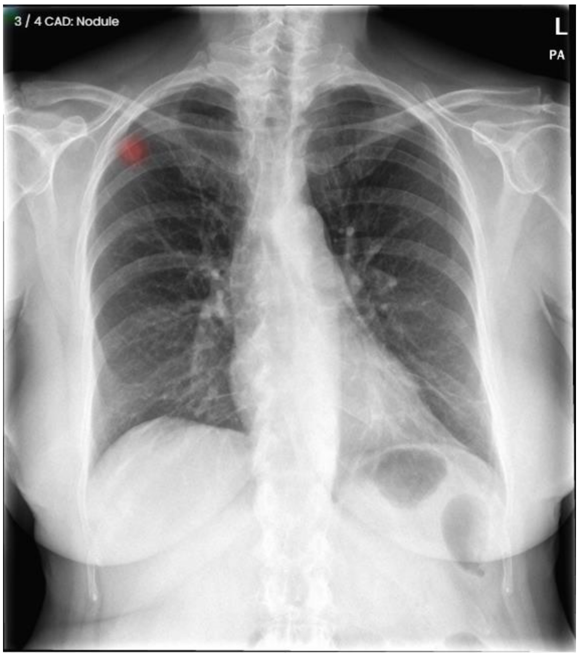
</td>
<td>AI-detected findings (ChestEye Quality)
Opacity Sensitive</td>
</tr>
<tr>
<td colspan="2">External radiologist assessment
Nodular opacity in the upper lobe of right lung.

Institution's radiologist assessment
Confirmed</td>
</tr>
</table>

Case 2

Original chest radiograph

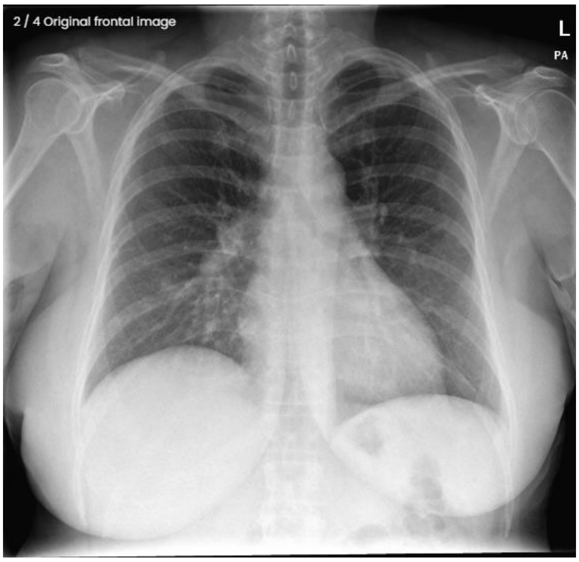

Original radiology report (automatic translation)
Chest X-ray Normal view of the heart, hili, lungs and mediastinum.

NLP labels
Normal

Overlay of AI-detected findings (in color)

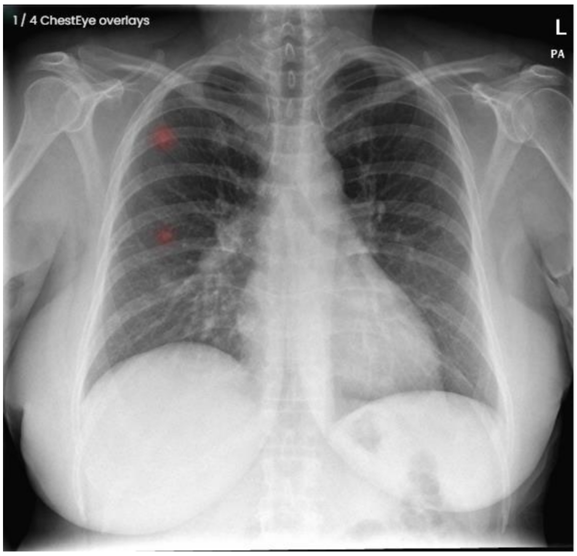

AI-detected findings (ChestEye Quality)
Nodule

External radiologist assessment
Small nodular opacities in the right lung.

Institution's radiologist assessment
Confirmed

Case 3

Original chest radiograph

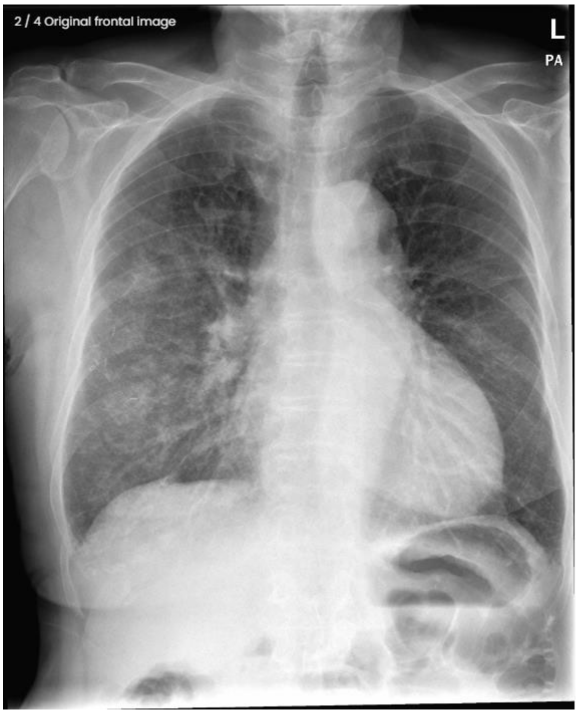

Original radiology report (automatic translation)

Propelled pulmonary circulation with slightly enlarged cor shadow (CTR XX/XX). No pleural effusion. No clear trim picture.

NLP labels

Enlarged_Heart

Overlay of AI-detected findings (in color)

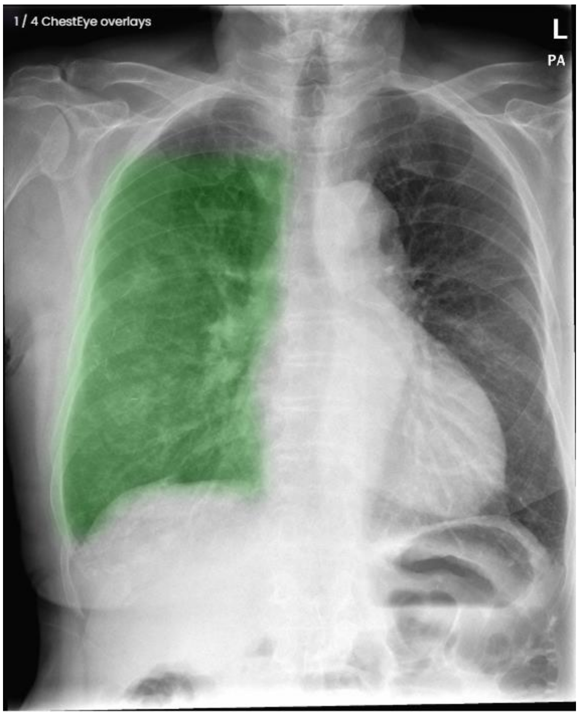

AI-detected findings (ChestEye Quality)

Consolidation

External radiologist assessment

Ill defined patchy opacities suspicious for alveolar involvement.

Institution's radiologist assessment

Confirmed

Case 4

Original chest radiograph

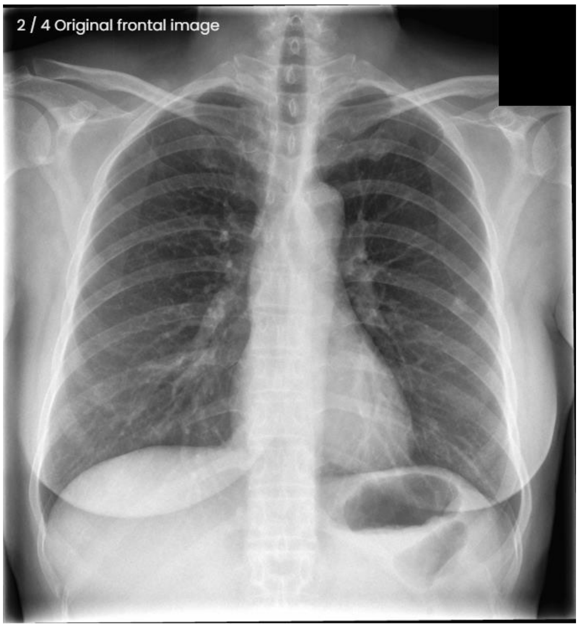

Original radiology report (automatic translation)

No previous research available for comparison. No consolidation. No need to suspect. Heart size and pulmonary vessel drawing within the norm. No pleural effusion.
Conclusion. No lung metastases detectable.

NLP labels

Normal

Overlay of AI-detected findings (in color)

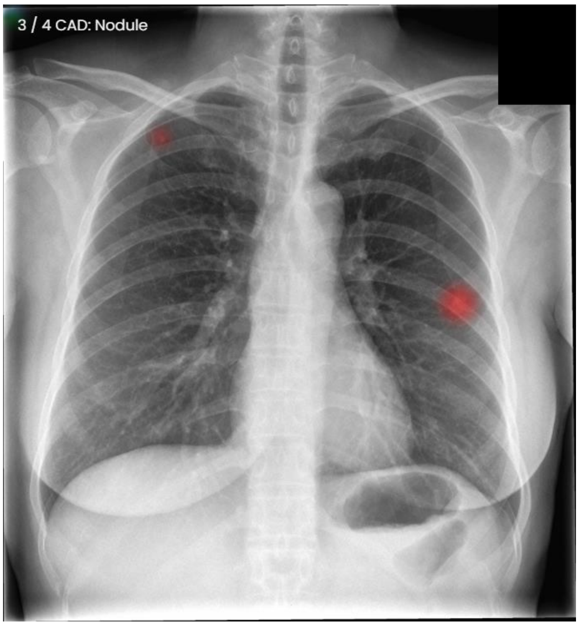

AI-detected findings (ChestEye Quality)

Nodule, Opacity Sensitive

External radiologist assessment

Suspicious small nodular opacity projected over inferior angle of scapula.

Institution's radiologist assessment

Confirmed

Case 5

Original chest radiograph

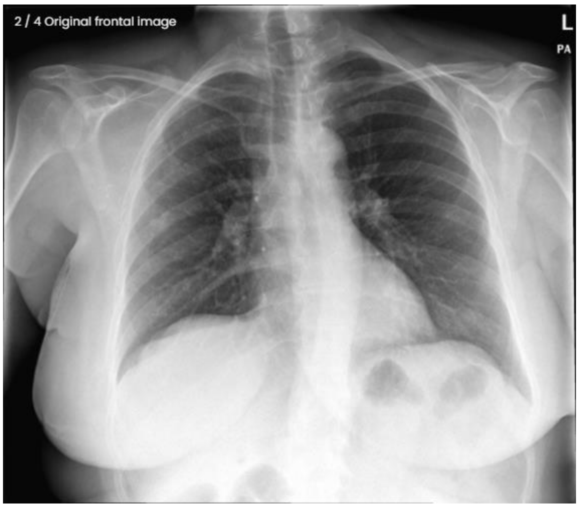

Original radiology report (automatic translation)
Normal view of the heart, hili, lungs and mediastinum.

NLP labels
Normal

Overlay of AI-detected findings (in color)

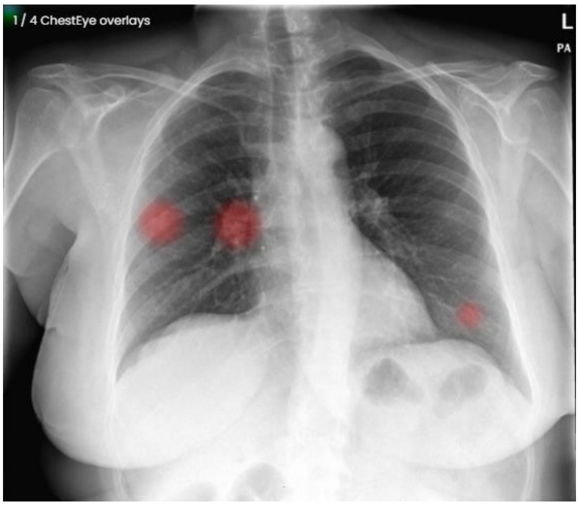

AI-detected findings (ChestEye Quality)
Opacity Sensitive

External radiologist assessment
Nodular opacity in right lung middle field.

Institution's radiologist assessment
Confirmed

Case 6

<table>
<tr>
<td>Original chest radiograph
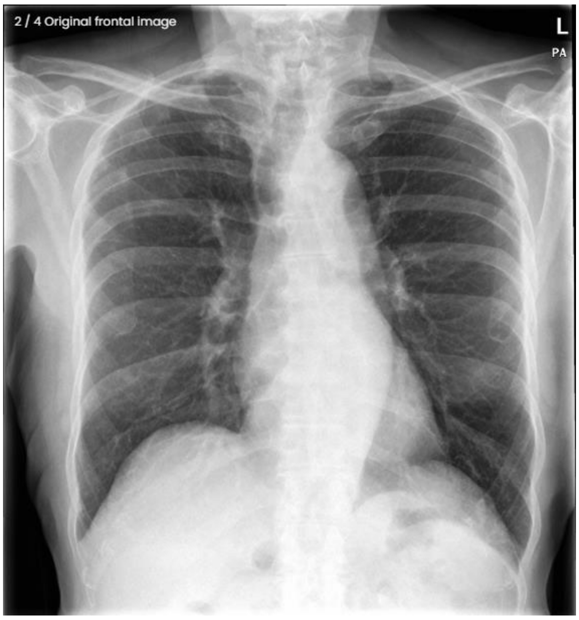
</td>
<td>Original radiology report (automatic translation)
For correlation PET-CT of XX/XX/XXXX. Normal cardiomediastinal silhouette. Normal hili and pulmonary vascular drawing. Well-delimited diaphragms. No nodules or consolidations.

Conclusion: No evidence of lymphadenopathy. No pulmonary abnormalities.

NLP labels
Normal</td>
</tr>
<tr>
<td>Overlay of AI-detected findings (in color)
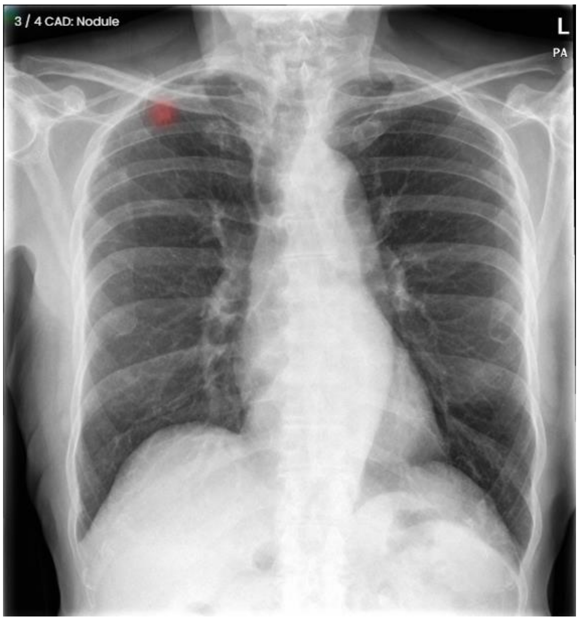
</td>
<td>AI-detected findings (ChestEye Quality)
Opacity Sensitive</td>
</tr>
<tr>
<td colspan="2">External radiologist assessment
Right apical nodular opacities.

Institution's radiologist assessment
Confirmed</td>
</tr>
</table>

Case 7

Original chest radiograph

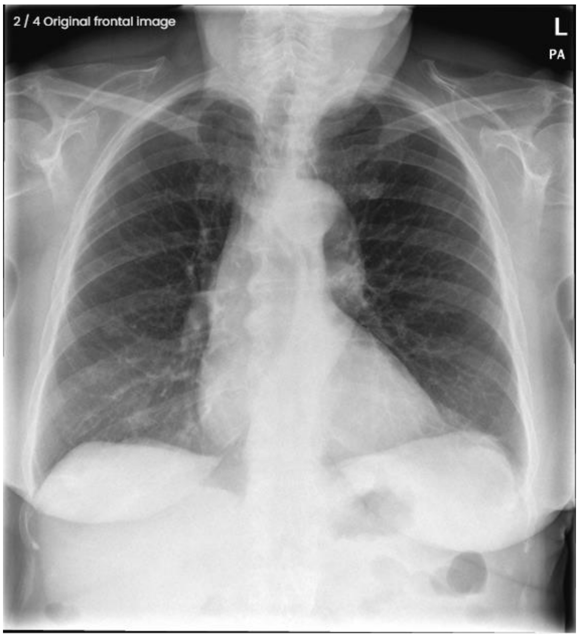

Original radiology report (automatic translation)
No suspected outbreaks detected.

NLP labels
Normal

Overlay of AI-detected findings (in color)

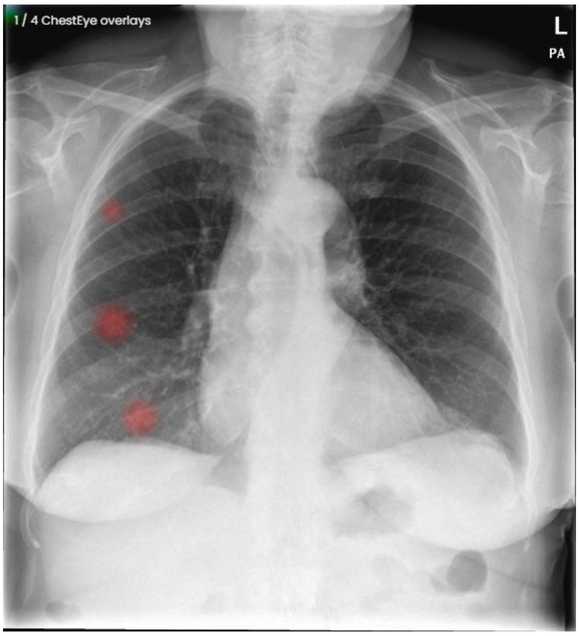

AI-detected findings (ChestEye Quality)
Opacity Sensitive

External radiologist assessment
Suspicious nodular opacity in the middle field of right lung.

Institution's radiologist assessment
Confirmed

Case 8

Original chest radiograph

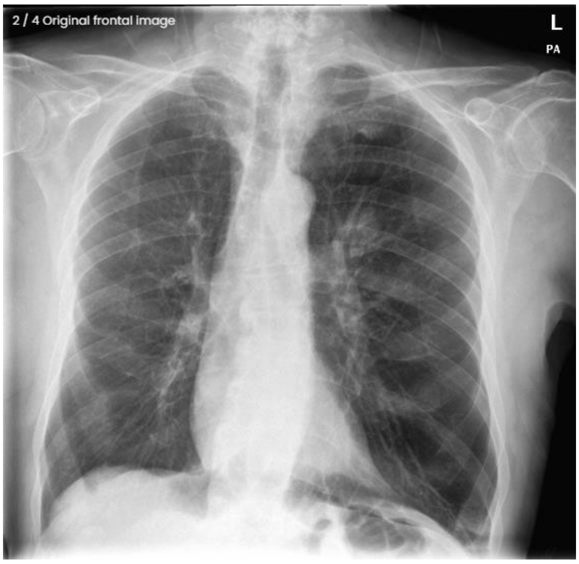

Original radiology report (automatic translation)

Flat diaphragm domes and reinforced front-back diameter to suit emphysema. Clear lung fields without pleural effusion or infiltrates, no pneumonitis picture. Slim heart figure. Normal hili with no evidence of lymphadenopathy. Slim mediastinum.

Conclusion. No pneumonitis picture or indications of infiltrates.

NLP labels

Normal

Overlay of AI-detected findings (in color)

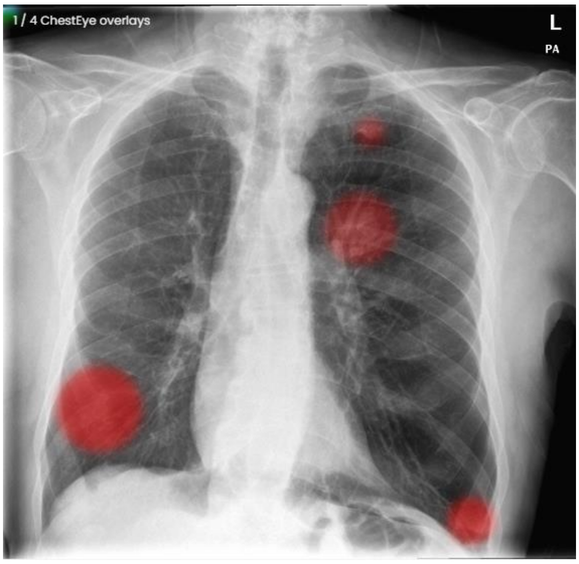

AI-detected findings (ChestEye Quality)

Nodule, Opacity Sensitive

External radiologist assessment

Nodular opacity in the projection above left hilum.

Institution's radiologist assessment

Confirmed

Case 9

Original chest radiograph

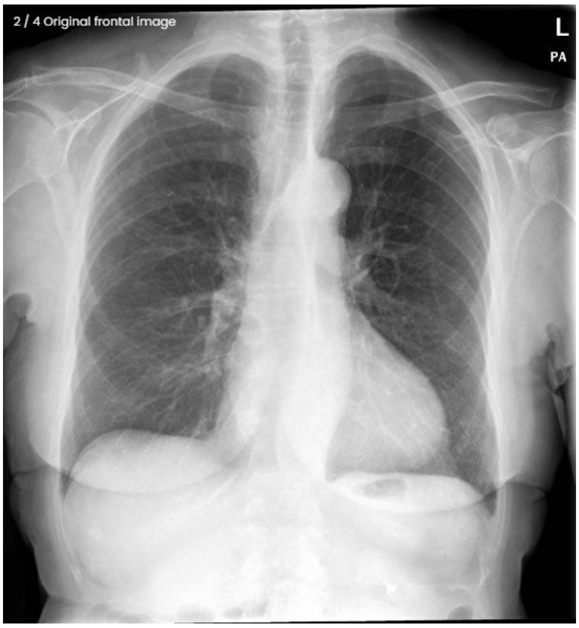

Original radiology report (automatic translation)

Compared to XX/XX/XXXX unchanged normal image of the heart, hili, lungs and mediastinum.

NLP labels

Follow_Up

Overlay of AI-detected findings (in color)

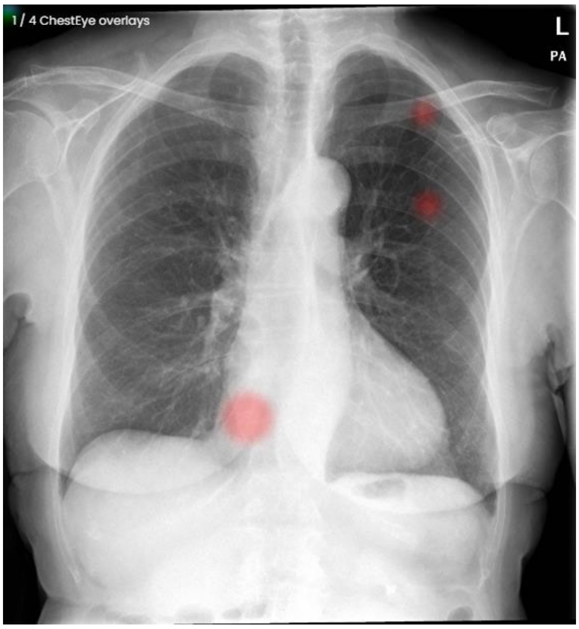

AI-detected findings (ChestEye Quality)

Opacity Sensitive

External radiologist assessment

Small suspicious nodular opacity in the upper-middle field of left lung.

Institution's radiologist assessment

Confirmed

Case 10

**Original chest radiograph**	**Original radiology report (automatic translation)**
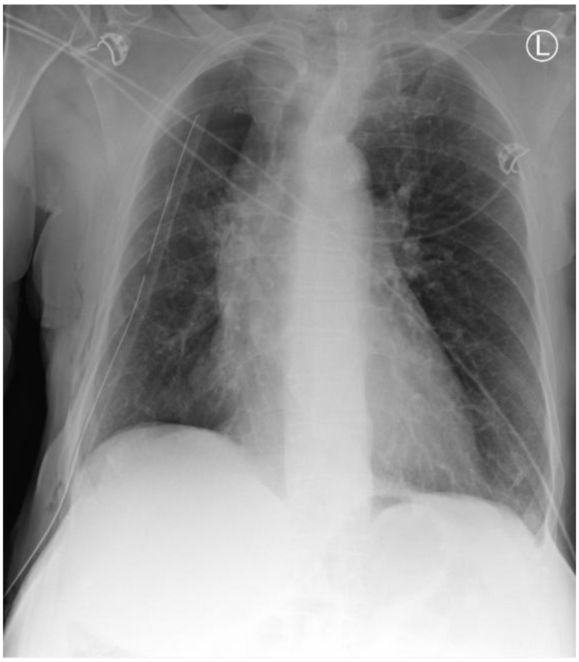	Compared to XX/XX/XXXX status after lobectomy right upper lobe with drain right in situ. No pneumothorax line visible. High standing right emerged. No trim picture. No moisture or other details. Minimal subcutaneous emphysema in the right flank.<br><br>**NLP labels**<br>Removed_Lung, Subcutaneous_Emphysema, Follow_Up
**Overlay of AI-detected findings (in color)**<br>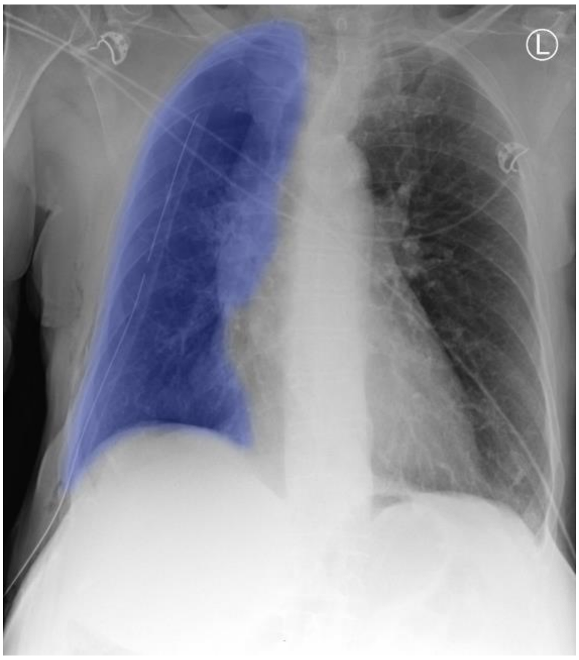	**AI-detected findings (ChestEye Quality)**<br>Pneumothorax
**External radiologist assessment**<br>Residual air in right pleural space suspected due to right apical lucency.<br><br>**Institution's radiologist assessment**<br>Confirmed	

Case 11

Original chest radiograph

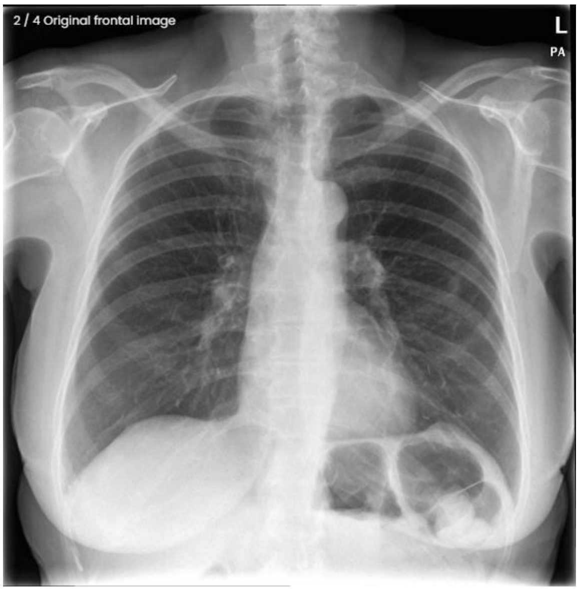

Original radiology report (automatic translation)

No old research available for comparison. Operation clips projecting over the left mom. Is the patient also known with a history of breast cancer? No relevant clinical information on this. No space-consuming processes, no lung metastases. Normal mediastinum and slender hili. No details in the skeleton.

Conclusion. No evidence of metastasis or second primary.

NLP labels

Normal

Overlay of AI-detected findings (in color)

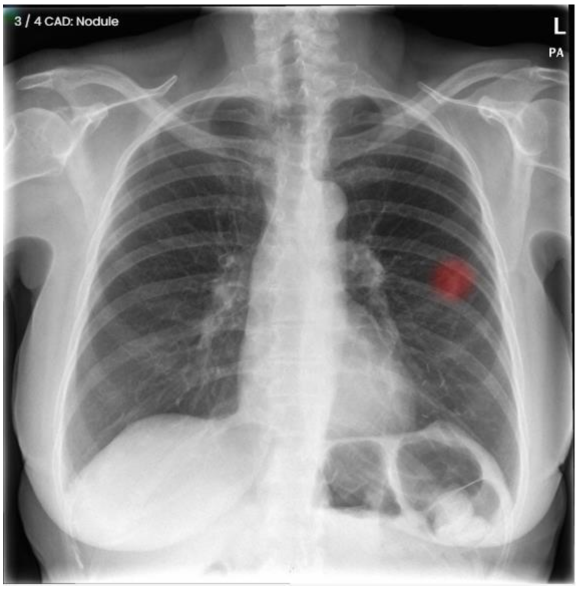

AI-detected findings (ChestEye Quality)

Opacity Sensitive

External radiologist assessment

Small nodular opacity in the middle field of left lung.

Institution's radiologist assessment

Confirmed

Case 12

Original chest radiograph

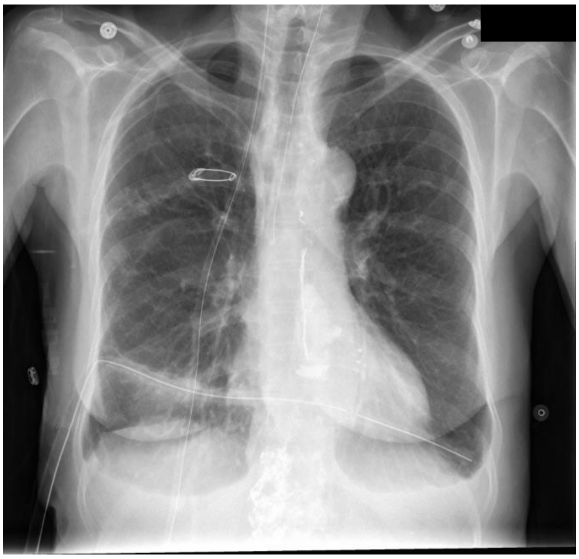

Original radiology report (automatic translation)

Siphon to mid-esophagus in situ. Chest drain where right crossing to left in situ, unchanged position. Unchanged rounded costophrenic sinus on both sides with trace of pleural effusion. Status after embolization thoracic duct with coils and lipiodol residues in situ.

Conclusion: Unchanged position of coils in status after thoracic duct embolization.

NLP labels

Endovascular_Coil, Follow_Up

Overlay of AI-detected findings (in color)

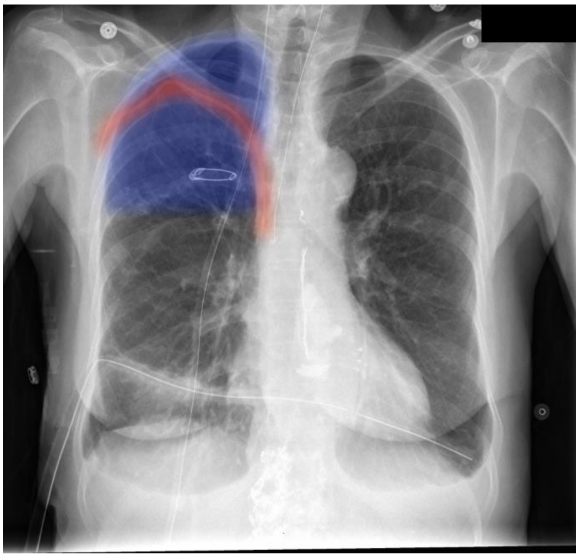

AI-detected findings (ChestEye Quality)

Pleural_Effusion, Pneumothorax, NG tube: Malposition, Catheter: Superior Vena Cava

External radiologist assessment

Small right sided apical pneumothorax.

Institution's radiologist assessment

Confirmed

Case 13

Original chest radiograph

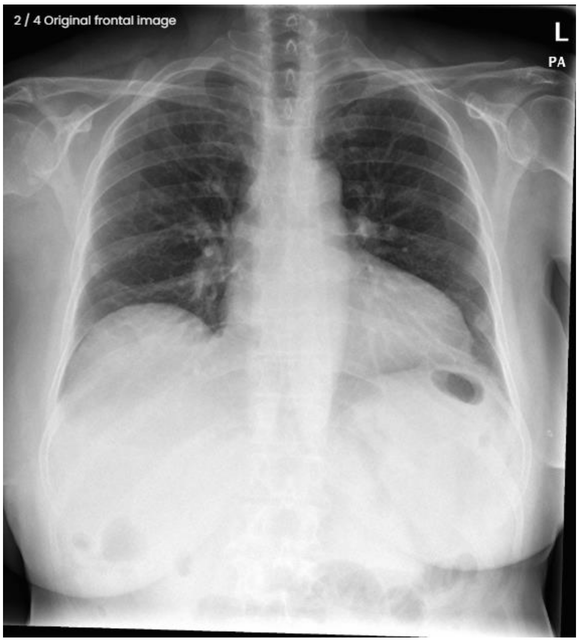

Original radiology report (automatic translation)
Moderate inspiratory position. Otherwise normal picture of the heart, hili, lungs and mediastinum. No moisture, stagnation or infiltrate formation.

NLP labels
Normal

Overlay of AI-detected findings (in color)

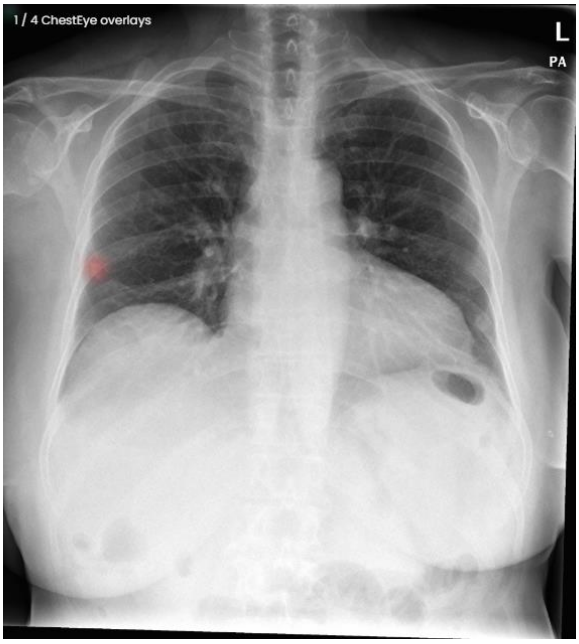

AI-detected findings (ChestEye Quality)
Nodule

External radiologist assessment
Small nodular opacity in the lower field of right lung.

Institution's radiologist assessment
Confirmed

Case 14

Original chest radiograph

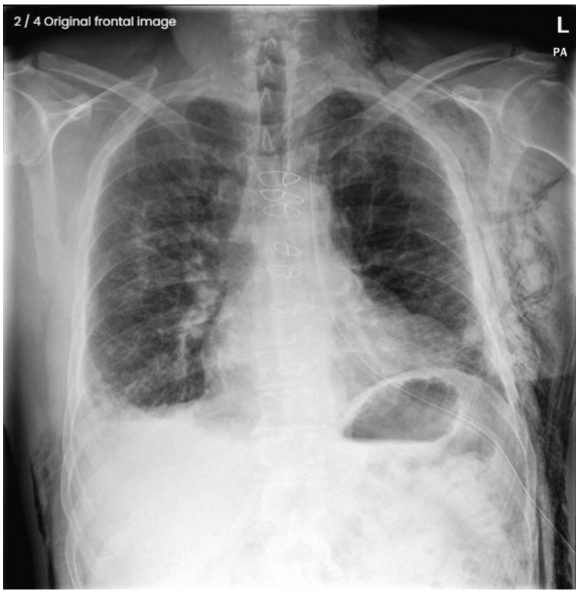

Original radiology report (automatic translation)
Compared to study 2 days earlier. Status after sternotomy. Thorax drain left in situ. Well deployed left lung. Progression of subcutaneous emphysema. Pleural effusion atelectasis right basal. Perihilair some compactions on the right.

Conclusion: Unchanged image of the left lung. Right pleural fluid atelectasis. Some perihilar infiltrates on the right.

NLP labels
Pleural_Effusion, Consolidation, Lobar_Collapse, Follow_Up

Overlay of AI-detected findings (in color)

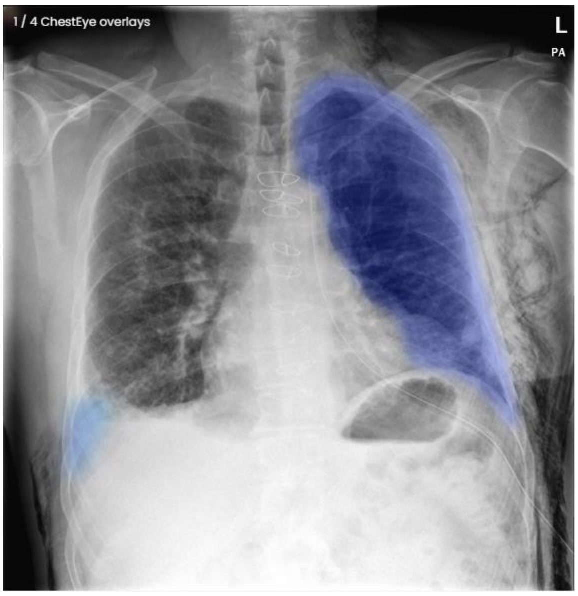

AI-detected findings (ChestEye Quality)
Pleural_Effusion, Pneumothorax

External radiologist assessment
Trace left apical pneumothorax suspected.

Institution's radiologist assessment
Confirmed

Case 15

**Original chest radiograph**<br>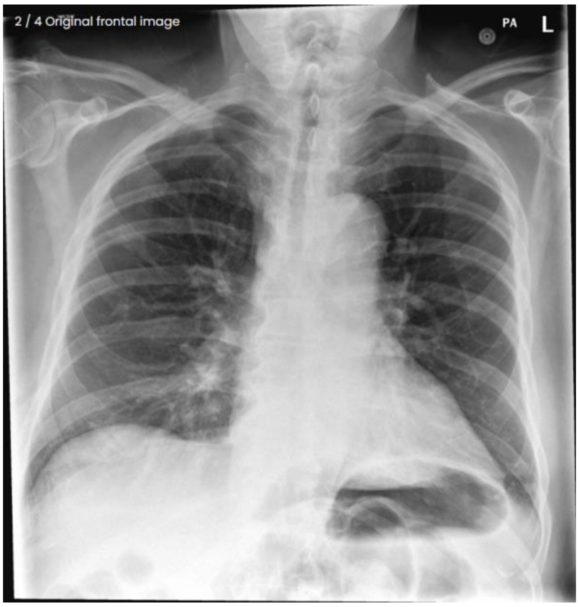 	**Original radiology report (automatic translation)**<br>No evidence of pneumothorax. No pleural effusion.<br><br>**NLP labels**<br>Normal
**Overlay of AI-detected findings (in color)**<br>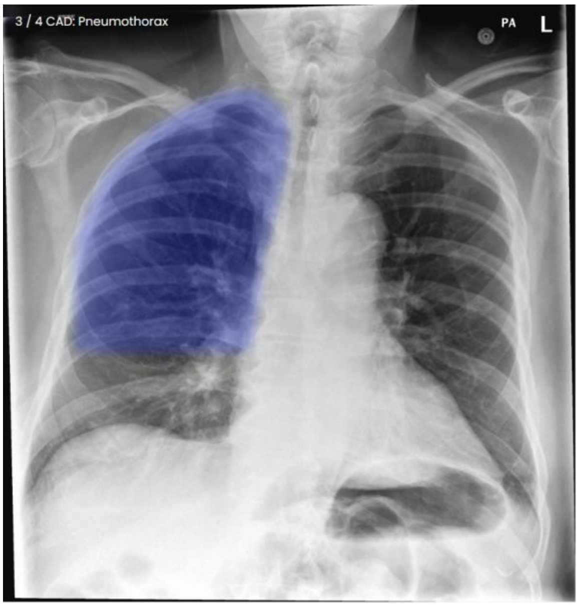 	**AI-detected findings (ChestEye Quality)**<br>Pneumothorax
**External radiologist assessment**<br>Small right apical pneumothorax cannot be reliably excluded.<br><br>**Institution's radiologist assessment**<br>Rejected - finding was not unequivocally present	

Case 16

Original chest radiograph

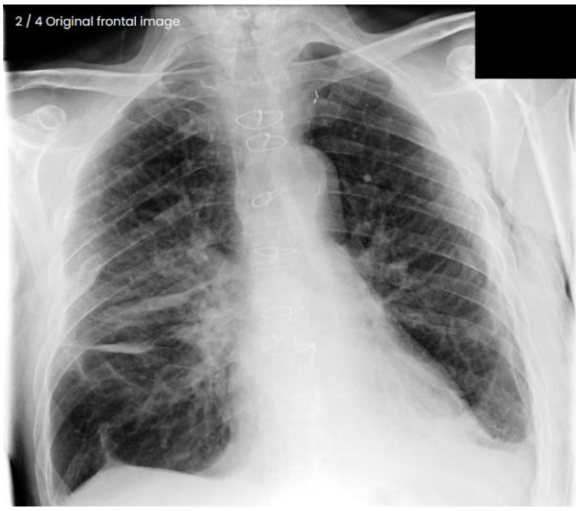

Original radiology report (automatic translation)

It is compared with XX/XX/XX. Sinus pleura not shown dorsally. Status after gastric tube removal. Status after sternotomy. Known break in the lower cercle thread. Known fibrotic changes on both sides. Bronchopathy especially retrocardial. Consolidated rib fracture on the right. Figure and lung vessel drawing within standard. Unchanged still slight perihilar consolidation right.

Conclusion: Unchanged still slight perihilar consolidation on the right. No signs of overfilling.

NLP labels

Consolidation, Follow_Up

Overlay of AI-detected findings (in color)

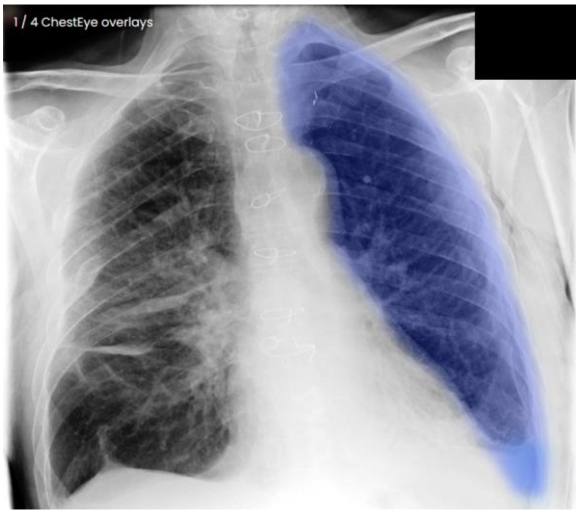

AI-detected findings (ChestEye Quality)

Pleural_Effusion, Pneumothorax

External radiologist assessment

Small left lateral pneumothorax and subcutaneous emphysema suspected.

Institution's radiologist assessment

Rejected - finding was not unequivocally present

Case 17

Original chest radiograph

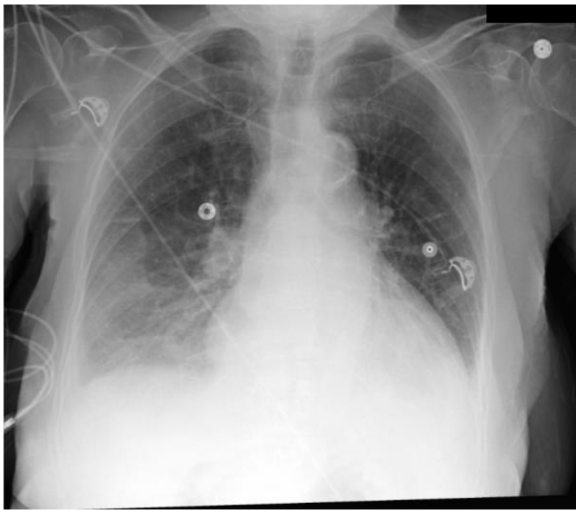

Original radiology report (automatic translation)

Recording from the day before for comparison. Slight decrease in the trapping image, no normalization yet.

NLP labels

Congestion, Follow_Up

Overlay of AI-detected findings (in color)

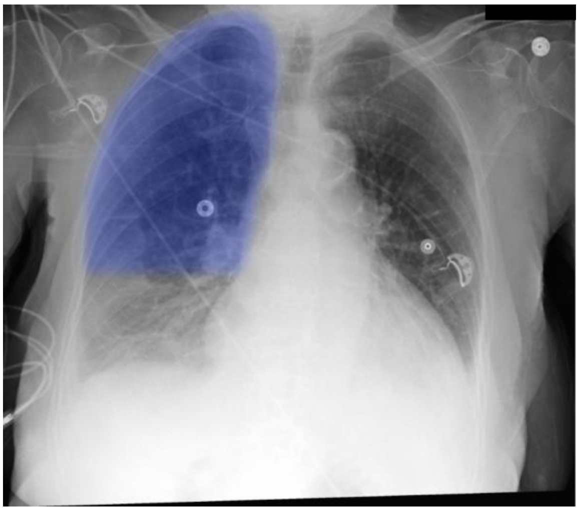

AI-detected findings (ChestEye Quality)

Pleural_Effusion, Pneumothorax

External radiologist assessment

Small right apical pneumothorax suspected.

Institution's radiologist assessment

Rejected - finding was not unequivocally present

Case 18

Original chest radiograph

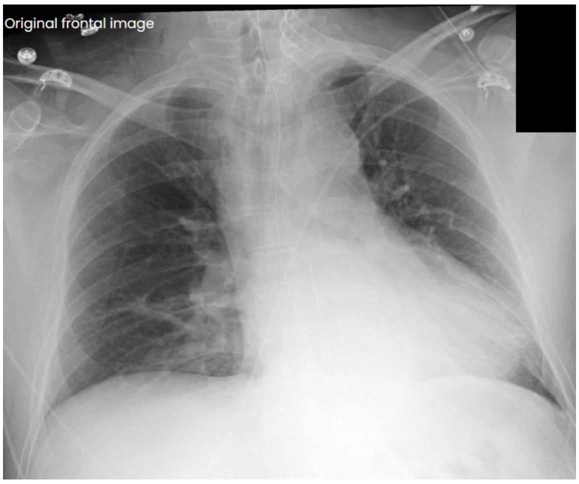

Original radiology report (automatic translation)

Compared to XX/XX/XXXX, the lines are unchanged. Also otherwise normal picture of heart, hili, lungs and mediastinum.

NLP labels

CV_Catheter_SVC_Placement, Follow_Up

Overlay of AI-detected findings (in color)

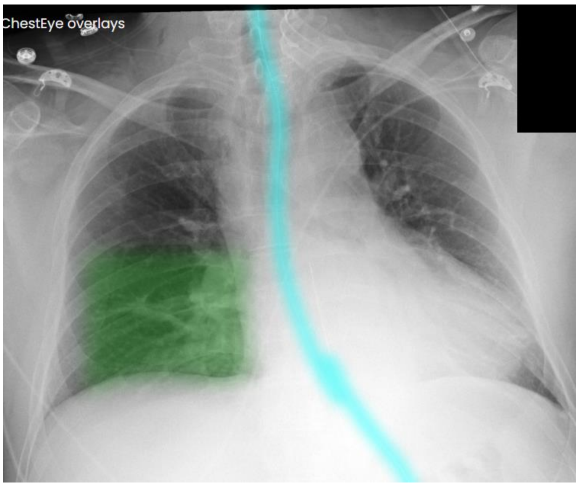

AI-detected findings (ChestEye Quality)

Consolidation, NG tube: Correct Position

External radiologist assessment

Linear opacity in right lung lower field, may be suggestive of plate atelectasis.

Institution's radiologist assessment

Rejected - finding was present but deemed not clinically relevant

Case 19

Original chest radiograph

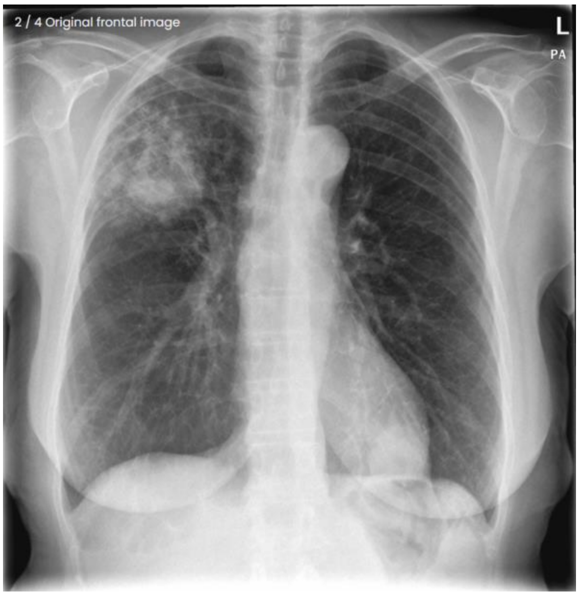

Original radiology report (automatic translation)

Chest X-ray after biopsy shows no pneumothorax. The patient was sent home in good condition, with instructions to report to any clinic that appeared.

NLP labels

Normal

Overlay of AI-detected findings (in color)

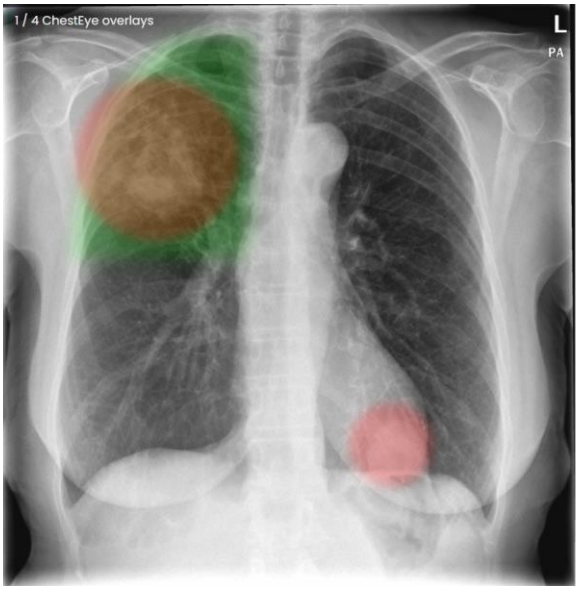

AI-detected findings (ChestEye Quality)

Nodule, Consolidation

External radiologist assessment

Consolidation in the right upper lobe suspicious for post-biopsy intrapulmonary hemorrhage.

Institution's radiologist assessment

Rejected - finding was known and unchanged in comparison to prior imaging

Abbreviations

AI: Artificial Intelligence; CV: central venous; NG: nasogastric; NLP: Natural Language Processing; SVC: superior vena cava
